# Dengue Virus NS5 Target Discovery: A Comprehensive in Silico Exploration of Novel Druggable Sites for Pan-Serotype Antiviral Design

**DOI:** 10.3390/ijms27125639

**Published:** 2026-06-22

**Authors:** João Trigueiro-Louro, Vanessa Correia, Inara dos Santos Ali, Bulbul Ahmed, Helena Rebelo-de-Andrade

**Affiliations:** 1 Antiviral Resistance Lab, Research & Development Unit, Infectious Diseases Department, Instituto Nacional de Saúde Doutor Ricardo Jorge, IP, Av. Padre Cruz, 1649-016 Lisbon, Portugal; 2 Host-Pathogen Interaction Unit, Research Institute for Medicines (iMed.ULisboa), Faculty of Pharmacy, Universidade de Lisboa, Av. Professor Gama Pinto, 1649-003 Lisbon, Portugal; 3 Serviço de Medicina, Hospital de São Francisco Xavier, Unidade Local de Saúde de Lisboa Ocidental (ULSLO), Estrada do Forte do Alto do Duque, 1449-005 Lisbon, Portugal; 4 School of Agriculture, Galgotias University, Greater Noida 203201, India

**Keywords:** dengue virus, RNA-dependent RNA polymerase, sequence conservation, structure-based druggability prediction, resistance-resilient antiviral targets, pan-serotype strategy

## Abstract

Dengue is the most common vector-borne viral disease worldwide, posing an increasing global health threat. Despite its high burden, no approved antiviral treatments or widely applicable vaccines exist, and patient management remains limited to supportive care, underscoring the urgent need for antiviral development. The NS5 protein is a prime antiviral target, owing to its crucial role in viral replication, high conservation across dengue virus (DENV) serotypes and lack of a human orthologue. We conducted a comprehensive sequence-to-structure analysis to identify conserved druggable regions within NS5, integrating large-scale sequence analysis with structural characterization across all four DENV serotypes. We identified four highly promising Consensus Druggable Pockets within the NS5 dimer—CDP1d, CDP3d, CDP5d and CDP12d—that overlap functionally critical regions, alongside 149 new potential hot spot residues. Domain-specific analysis revealed that MTase offers more densely conserved targets, whereas RdRp provides broader druggable surfaces, revealing complementary features for pharmacological modulation. Several identified pockets spatially overlap known inhibitor binding sites, and preliminary docking analyses support their capacity to accommodate small molecules, reinforcing their therapeutic relevance as candidate targets. Collectively, these findings provide a robust framework for the rational design of pan-serotype anti-DENV NS5 antivirals with an enhanced barrier to resistance.

## 1. Introduction

Dengue is the most prevalent vector-borne viral disease, currently endemic in over 100 countries across tropical and subtropical regions, with increasing reports of autochthonous transmission in temperate areas, including mainland Europe [1,2]. Drivers such as climate change, urbanization, and intensified global travel have expanded *Aedes* vectors beyond intertropical zones and intensified transmission dynamics [3,4]. An estimated half of the world’s population is at risk, with 100–400 million infections annually, of which approximately 60 million are symptomatic and up to 40,000 are fatal [1,5]. Reflecting this growing impact, the World Health Organization (WHO) has listed dengue among its top 10 public health threats since 2019 and, more recently, as a priority pathogen for epidemic and pandemic preparedness [6,7]. Dengue virus (DENV), a member of the *Flaviviridae* family, comprises four antigenically distinct yet genetically related serotypes (DENV1–DENV4), of which DENV1 and DENV2 circulate most widely worldwide [4,8].

To date, no clinically approved antivirals or widely applicable vaccines for dengue are available and clinical management remains limited to supportive care, focused on symptomatic relief and fluid balance [1,9]. Despite decades of effort, antiviral development remains largely stalled at the preclinical stage [10]. Numerous compounds with promising in vitro activity have failed to progress owing to poor physicochemical and pharmacokinetic properties, a low genetic barrier to resistance, or in vivo toxicity (e.g., NITD-448, BP13944, NITD-008) [9,11,12,13]; and repurposed drugs that reached clinical trials (balapiravir, chloroquine, lovastatin, and celgosivir) did not significantly reduce viremia or improve clinical outcomes [14]. Direct-acting antivirals (DAAs), which target key stages of the viral replication cycle through interaction with viral proteins, are widely regarded as more potent and selective than host-directed therapies [15]. Among these, non-structural (NS) proteins are particularly attractive targets: expressed exclusively in infected cells, they are exposed to lower immune-mediated selective pressure than structural proteins, which contributes to their higher sequence conservation, lower mutational variability and reduced likelihood of resistance development, features that also support their potential for broad-spectrum strategies across related viruses [16].

**Figure 1:**
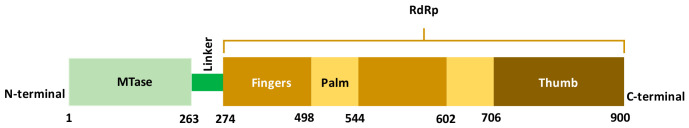
Schematic representation of the DENV NS5 protein structure. The N-terminal Methyltransferase (MTase) and C-terminal RNA-dependent RNA Polymerase (RdRp) domains are connected by a linker region. The three RdRp subdomains—Fingers, Palm, and Thumb—are also depicted. Residue numbering follows DENV2. Adapted from Osawa et al. [18].

Non-structural protein 5 (NS5) is the largest and most conserved NS protein of DENV, sharing approximately 75% sequence identity among the four serotypes [9]. This multifunctional enzyme comprises an N-terminal methyltransferase (MTase) and a C-terminal RNA-dependent RNA polymerase (RdRp), connected by a short linker region (residues 264–273) that regulates NS5 conformational dynamics and modulates MTase–RdRp interactions [17] (Figure 1).

The MTase domain (residues 1–263, DENV2 numbering) catalyses methylation of the viral RNA cap (5′ capping), protecting the genome from degradation, enhancing its interaction with the host translational machinery and enabling immune evasion [17]. The RdRp domain (residues 274–900) constitutes the core enzymatic machinery for the de novo synthesis of the viral RNA genome [18,19]; it adopts the canonical right-hand architecture comprising the Fingers, Palm and Thumb subdomains, which harbour seven conserved sequence motifs (A–G) essential for RNA template binding, nucleoside triphosphate (NTP) incorporation, metal-ion coordination, and catalysis [20]. Beyond its enzymatic activities, NS5 engages with the NS protein 3 (NS3), host cellular factors and the 5′ stem-loop A structure to promote viral replication and suppress host antiviral responses [9]. Critically, both NS5 enzymatic activities are absent in human cells, underscoring NS5 as a highly attractive target for antiviral intervention with minimal off-target effects [10,21].

A substantial preclinical portfolio targets NS5, comprising over 40 compounds with reported in vitro and/or in vivo antiviral activity (Table S1). However, the development of most of these candidates has not been guided by structure-based strategies specifically targeting NS5. Many are natural products identified through exploratory phytochemical screening of medicinal or native plant extracts, while others have arisen from drug repurposing efforts developed against human immunodeficiency virus type 1 (HIV-1), filoviruses, severe acute respiratory syndrome coronavirus 2 (SARS-CoV-2), and particularly hepatitis C virus (HCV). Several candidates have been identified through high-throughput screening strategies targeting either viral replication or specific NS5-associated functions, such as the GTP-binding activity of MTase domain (e.g., BG-323) [22]. Only a limited number of candidates (6 candidates) have been rationally designed or screened to target defined structural features of NS5, and even in these cases the selection of target sites was not informed by a comprehensive assessment of sequence conservation. The only reported exception concerns benzothiophene derivatives targeting a conserved Triple-D motif within the active site of a consensus RdRp flavivirus structure [23]; however, this analysis was restricted to the core residues and did not account for the broader binding interface (Table S1).

Together, these observations reveal two critical limitations in current anti-DENV drug discovery: (1) the predominance of candidates lacking a structure-based design and (2) the absence of integrative approaches combining structural targeting with comprehensive sequence conservation analysis of targetable regions. Addressing these shortcomings is essential, particularly given that inadequate target validation and suboptimal drug-like properties account for up to 60% of clinical failures [16].

In this context, the present study aims to identify and map conserved druggable regions within DENV NS5 by integrating large-scale sequence conservation analysis of globally circulating viruses with structure-based druggability assessments derived from available crystallographic data. By establishing a unified framework, our work provides a robust foundation for guiding the rational design of next-generation, pan-serotype DENV antivirals with enhanced resistance resilience.

## 2. Results and Discussion

### 2.1. NS5 Length and Underlying Variations

Analysis of 4127 NS5 protein sequences from globally circulating human-infecting DENV revealed that the protein most frequently comprises 900 amino acids (aa) (58.5%), consistent with the canonical length reported in the literature [17]. Variation in sequence length was observed across serotypes and in a limited number of individual strains, ranging from 896 to 901 aa. This variability results from minor insertion-deletion events (indels), predominantly involving single-aa deletions. Five length variant types (LVTs) were identified alongside the predominant 900-aa form (Table 1). Among these, the 899-aa variant was the most prevalent (41.4%), whereas the remaining variants (896, 897, 898, and 901 aa) were exceedingly rare (0.00024%). Inspection of GenBank annotations confirmed that the indels underlying these low-frequency variants are genuine and not sequencing artifacts. To the best of our knowledge, these NS5 LVTs have not been previously reported.

DENV1 NS5 is distinct from the other serotypes in being one aa shorter (899-aa LVT), due to a deletion at position 639 within the RdRp Palm subdomain. Additional deletions give rise to the three LVTs identified in DENV1: a 896-aa variant with a three-residue deletion in the MTase Core subdomain (residues 55–222) [24]; and the 897- and 898-aa variants associated with single-residue deletions in the RdRp domain, including the NS3 helicase-interacting region (residues 320–342) [25] (Table 1). In DENV2, the 899-aa LVT arises from single-residue deletions located either in the MTase Core (LT1) or the RdRp Fingers (LT2) subdomains. For DENV3, only two strains exhibited deviations from the canonical 900-aa length: an 899-aa variant arising from a single-residue deletion in the RdRp domain; and, a 901-aa variant caused by a single-residue insertion within motif B of the RdRp Palm subdomain (residues 598–614) [24]. By contrast, all DENV4 NS5 sequences retained the canonical length of 900 aa.

No consistent positional pattern was observed for the indels underlying the distinct LVTs, which were distributed throughout the NS5 protein (excluding the 899-aa form in DENV1, which represents the wild-type length in this serotype). Likewise, no clear temporal or geographic clustering was evident, as these LVT-containing variants displayed a broad range of geographic locations and time periods (Table 1). The only exception was observed for the 896- to 898-aa variants in DENV1, all of which were from 2007. The absence of discernible patterns, combined with their very low prevalence, suggests that these indels are unlikely to be selectively advantageous. Rather, they may reflect neutral or even deleterious variations that could impair NS5 function or structural integrity, thereby reducing viral fitness and limiting their persistence in circulating viral populations. Notably, one of the deletions associated with the DENV1 897-aa LVT occurs at residue 338, which lies within the NS5-NS3 helicase interaction interface, which is critical for stabilizing the viral replication complex and coordinating RNA unwinding and synthesis [25]. In addition, the insertion responsible for the DENV3 901-aa LVT falls within the conserved motif B of the RdRp domain (residues 612–613), a region critical for polymerase activity [17]. It is therefore reasonable to consider that variations within these key functional regions may compromise replication efficiency. However, further experimental studies are needed to clarify the precise impact of LVT-associated indels on NS5 activity and viral replication dynamics.

### 2.2. Sequence Conservation Profiles

**Figure 2:**
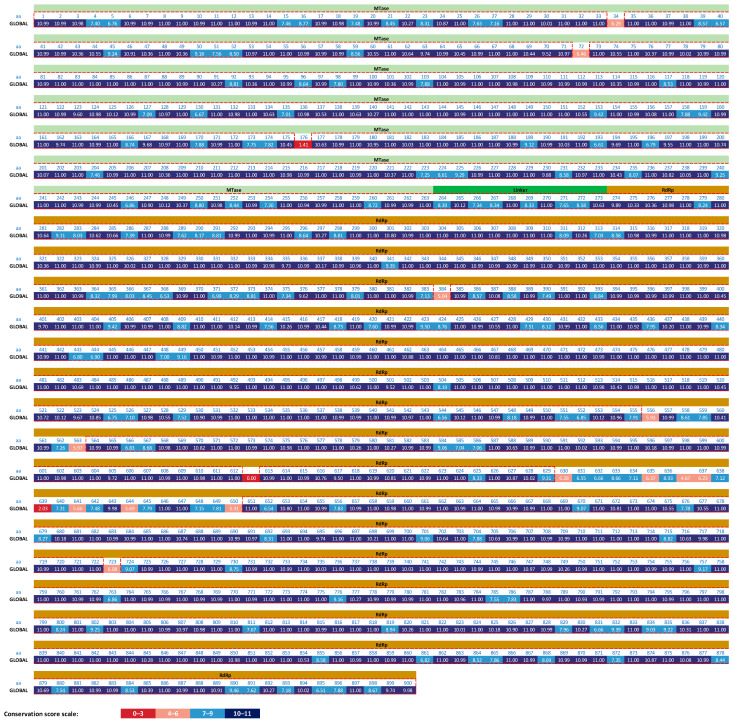
Residue-specific global conservation profile of the DENV NS5 protein. Functional domains are highlighted in light green (MTase) and light brown (RdRp), with the linker region shown in green. A color-coded scheme from red to blue was used to display the different conservation categories: red—highly variable residues (0–3); pink—variable residues (4–6); light blue—conserved residues (7–9); and, dark blue—highly conserved residues (10–11). The conservation score for each residue is displayed beneath its position in white. Conserved clusters (≥10 consecutive conserved residues) are indicated by dashed red lines.

NS5 is highly conserved across DENV serotypes, with 79.8% of residues classified as highly conserved (score 10), 18.4% as conserved (score 7–9), 1.4% as variable (score 4–6), and only 0.3% as highly variable (score 0–3). Residue-specific conservation scores mapped onto the protein architecture are shown in Figure 2. Conservation profiles were largely uniform across domains. The MTase domain comprised 81.4% highly conserved residues, 17.5% conserved residues, and 1.1% variable or highly variable residues; and the RdRp domain showed comparable values, with 79.7% highly conserved, 18.2% conserved, and 2.1% variable or highly variable residues. The RdRp domain is only marginally less conserved than the MTase domain, which is consistent with the predominance of LVT-associated indels in this domain (Section 2.1). Despite being the most functionally critical domain, the RdRp may be under slightly stronger selective pressure than the MTase domain, particularly in regions outside the catalytic core, potentially contributing to its slightly lower conservation.

In addition to individual residues, clusters of conserved sites—defined as regions comprising at least 10 consecutive residues with conservation scores ≥ 7—were identified across NS5. In total, nine such clusters were detected, with three located in the MTase domain, five in the RdRp domain, and one spanning both domains (Table S2). Cluster lengths varied widely (17 to 207 aa), with a general tendency toward large conserved stretches, which may enhance their relevance as potential druggable targets, particularly where structural and physicochemical properties favour drug binding.

Only two residues, T176 and N639, were classified as highly variable in the global NS5 protein (Figure 2). However, this classification arises from cross-serotype alignment artefacts, as both positions correspond to sites affected by serotype-specific deletions (Figure S2). Specifically, DENV1 and DENV3 NS5 exhibit a deletion at position 176, while DENV3 NS5 carries an additional deletion at position 639. In contrast, these two residues are conserved or highly conserved in DENV2 and DENV4. A similar pattern was observed between residues 612 and 613 within the RdRp Palm subdomain (Figure 2), where the apparent highly variability arises from a single insertion in one DENV3 strain (Section 2.1; Figure S2). Twelve residues exhibited a variable profile in the global NS5 protein, including two within the MTase domain (M34 and K72) and the remainder within the RdRp domain (F384, I556, L563, I630, E635, E637, E641, R644, E650 and G723) (Figure 2). None of these positions overlap with known functional or structurally critical regions, consistent with the strong conservation typically observed at sites essential for NS5 activity. Notably, most variable residues in RdRp cluster within the Palm subdomain, specifically in the region spanning residues 630 to 650. Although this region does not correspond to core catalytic motifs, its variability contrasts with the overall structural conservation characteristic of the Palm subdomain [26]. This pattern suggests that this segment (630–650) is unlikely to represent a robust target for antiviral design.

The overall global conserved profile observed for DENV NS5 is consistent with the high degree of conservation previously reported for this protein [27]. A detailed distribution of global conservation scores across NS5 protein is provided in Figure S1.

Serotype-specific analysis revealed a highly conserved NS5 protein across all four serotypes, with 94.1%–98.9% of residues classified as highly conserved and only minor proportions exhibiting variability (0.1–1.3%) (Table 2). Among them, DENV3 displays the highest degree of conservation, with nearly all residues classified as conserved and highly conserved. The only deviation was the additional residue between positions 612 and 613, resulting from an insertion detected in one DENV3 strain (Section 2.1), which was classified as highly variable. Conversely, DENV2 was slightly less conserved than the other serotypes, exhibiting the lowest proportion of highly conserved residues (94.1%) and a higher fraction of variable positions (1.3% each). This pattern may be attributed to its greater genetic diversity, reflected by a higher number of genotypes—6 DENV2 genotypes, compared with 2 to 5 for the other serotypes—that potentially contribute to its broad geographic distribution and global prevalence [8]. To the best of our knowledge, these subtle differences in conservation across DENV serotypes have not been previously reported. Residue-level conservation profiles for each serotype are shown in Figure S2.

The extensive conservation of DENV NS5 across and within serotypes reinforces its relevance as a candidate for antiviral targeting, for which subsequent structural analyses of druggability and spatial pocket distribution will be pivotal for guiding design efforts. 

### 2.3. Structure-Based Druggability Analysis

The NS5 of flaviviruses assembles into distinct oligomeric forms, with the dimer representing the predominant conformation in DENV, and therefore considered as the protein’s potentially active state [28,29]. In this context, NS5 dimers have been proposed to coordinate RNA replication within the viral replication complex, as the two protein domains are physically linked, in contrast to the monomeric structure where the MTase and RdRp active sites face away from each other and do not directly interact to perform this function [28]. Nonetheless, the monomeric form (particularly its RdRp domain) has been widely investigated, owing to its central role in viral RNA replication and its accessibility for structural and biochemical studies. Notably, the monomer is not merely an experimental construct. Recent cryo-EM structures capture DENV NS5 in its monomeric form bound to RNA, specifically to the 5′ stem–loop A (SLA) promoter RNA, to protein partners such as the NS3 helicase in RNA-elongating NS5-NS3 complexes, and to host factors like human STAT2 [18,30,31]. Importantly, investigating both dimeric and monomeric states enable a more comprehensive characterization of the protein’s druggability landscape across its functional spectrum. Therefore, we here performed a comprehensive in silico analysis encompassing both the full-length NS5 dimer and the monomeric RdRp domain. This dual-conformation approach is vital, as NS5 oligomerization may influence ligand accessibility and binding site architecture, thereby offering opportunities to design inhibitors that could target either specific conformational states or disrupt the oligomerization process itself.

#### 2.3.1. NS5-RdRp Monomer

**Figure 3:**
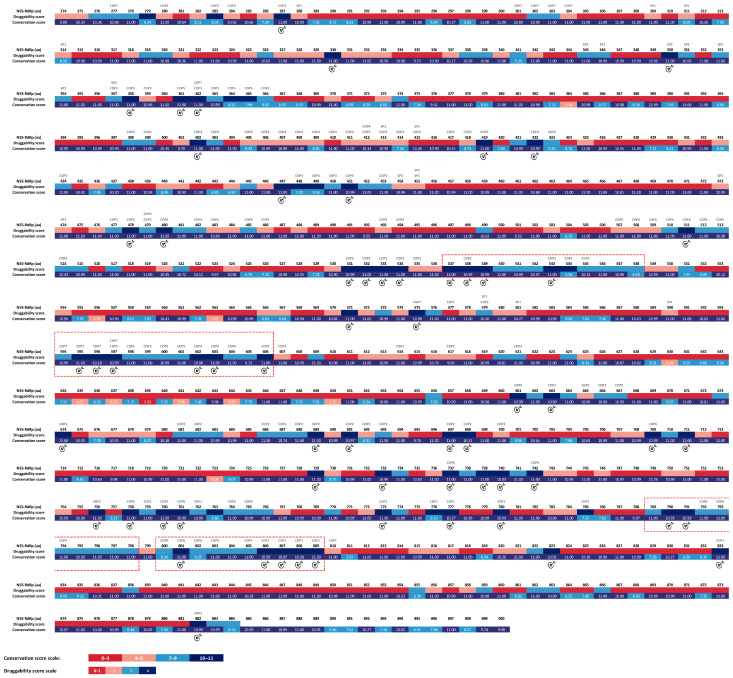
Linear alignment of NS5-RdRp druggability and global conservation scores. The figure shows a linear representation of the NS5-RdRp domain, with druggability scores correlated to global conservation at each residue. Predictions were made using a consensus approach from the MOE SiteFinder (MOE SF) and DoGSiteScorer (DGSS) tools. The color-coded scale progresses from red to blue, representing an increase in both conservation (from highly variable, 0–3, to highly conserved, 10–11) and druggability score (from very low, 0–1, to very high, 4). The putative Top-Ranked Hot Spots (T-RHS) are marked with a target symbol, and potential conserved druggable clusters are highlighted with dashed red lines. The positions of the potential Consensus Druggable Pockets (CDPs) and small consensus pockets (SPs) are indicated above the alignment.

The linear representation of the NS5-RdRp domain in Figure 3 enables direct comparison between druggability and global conservation scores. This integrative analysis identified 62 putative Top-Ranked Hot Spots (T-RHS), detailed in Table S3. The convergence of high conservation and favourable druggability at these sites makes them promising targets for broad-spectrum antiviral design with expected enhanced resistance resilience.

The identified 62 potential T-RHS were distributed across the three RdRp subdomains, with lower density in the Fingers subdomain (12 T-RHS, 6.6%) and comparable representation in the Palm (19 T-RHS, 12.7%) and Thumb (25, 12.8%) subdomains. Despite similar densities in the Palm and Thumb regions, the Palm subdomain emerges as the most promising for antiviral targeting, owing to its central role as the catalytic core of the polymerase, where key motifs essential for viral RNA synthesis reside. Hot spots within this region are therefore more likely to map to structurally and functionally constrained sites, enhancing their suitability for structure-based drug design.

Through comparative analysis of MOE-SiteFinder (MOE-SF) and DoGSiteScorer (DGSS) results across the two NS5-RdRp structures, seven potential Consensus Druggable Pockets (CDPs; ≥14 residues) and two potential small consensus pockets (SPs; 10–14 residues) were identified within the RdRp monomer. Residue composition and T-RHS content are provided in Table S4. The CDPs were distributed as follows: CDP1 and CDP6 in the Thumb subdomain (17 and 22 residues, respectively); CDP2 in the Palm subdomain and CDP4 spanning the Fingers and Palm subdomains (26 residues each); and CDP3, CDP5, and CDP7 in the Fingers subdomain (23, 17, and 15 residues, respectively). These CDPs represent promising targets for pharmacological modulation. Among them, CDP2 is the most suitable candidate, combining a high druggability score (0.68), substantial size (26 residues), and location within the functionally critical Palm subdomain.

#### 2.3.2. Full-Length NS5 Dimer

**Figure 4:**
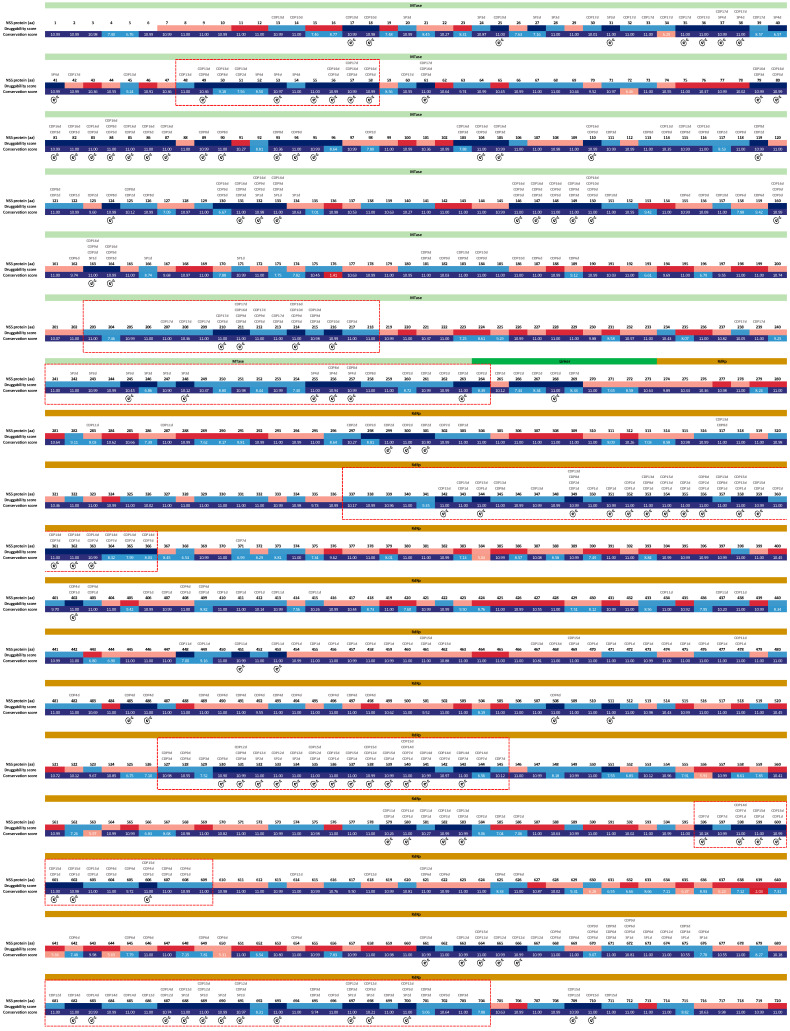

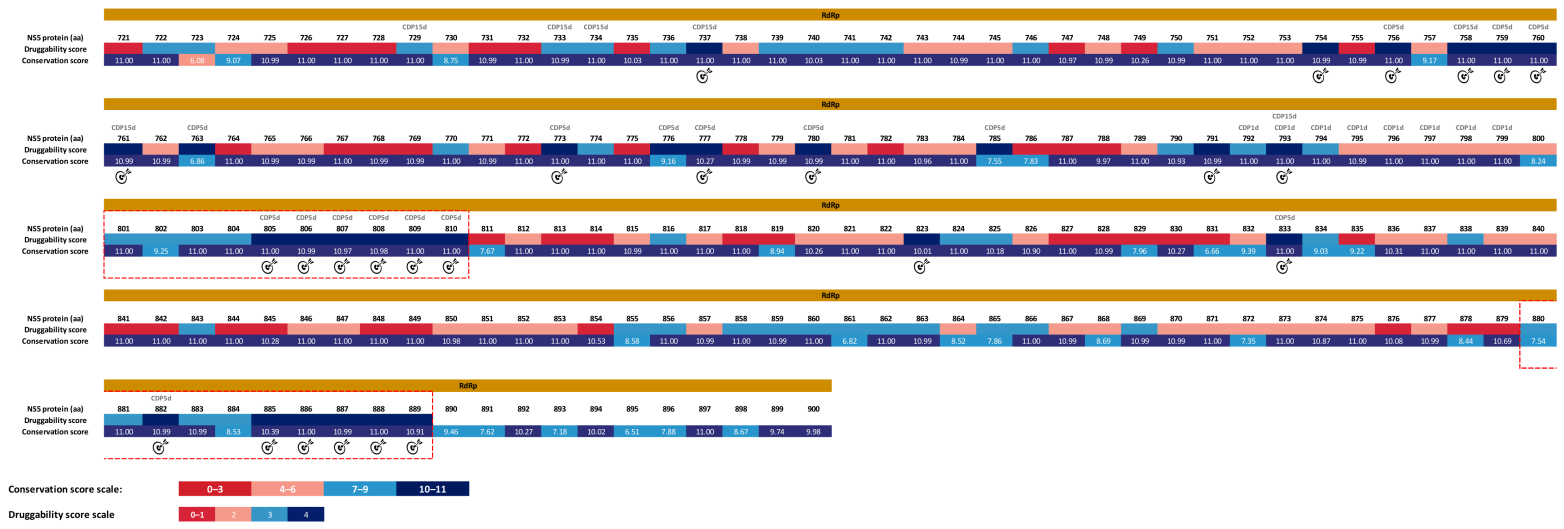
Linear alignment of druggability and global conservation scores along the full-length DENV NS5 dimer. The figure shows a linear representation of the NS5 dimer, with druggability scores correlated to global conservation at each residue. Predictions were made using a consensus approach from the MOE-SiteFinder (MOE-SF) and DoGSiteScorer (DGSS) tools. The color-coded scale progresses from red to blue, representing an increase in both conservation (from highly variable, 0–3, to highly conserved, 10–11) and druggability score (from very low, 0–1, to very high, 4). The putative Top-Ranked Hot Spots (T-RHS) are marked with a target symbol, and potential conserved druggable clusters are highlighted with dashed red lines. The positions of the potential Consensus Druggable Pockets (CDPs) and small consensus pockets (SPs) are indicated above the alignment.

A direct comparison of NS5 dimer druggability and global conservation scores revealed 149 putative T-RHS, visualized in the linear representation of the full-length protein (Figure 4) and detailed in Table S3. The MTase domain showed the highest hot spot density (56 T-RHS, 21.3%), followed by the RdRp domain (92 T-RHS, 14.7%), with only a single T-RHS in the linker region (0.1%). The paucity of hot spots in the linker, together with its intrinsic flexibility, makes it an unlikely target for antiviral intervention. Within the RdRp domain, the Palm subdomain retained the highest density (33 T-RHS, 22.0%), consistent with the monomeric state, followed by the Fingers (31 T-RHS, 17.0%) and Thumb (28 T-RHS, 14.4%) subdomains. This distribution reinforces the Palm subdomain as a promising target region, reflecting its central catalytic role and functional conservation, while the high hot spot density of the MTase domain identifies it as a second focus for structure-based design.

A total of 17 potential consensus pockets (≥14 residues) and 4 small consensus pockets (SPds; 10–14 residues) were identified in the NS5 dimer. Of the 17, only 12 met all classification criteria (including a druggability score ≥ 0.4) and were designated candidate Consensus Druggable Pockets (CDPds; CDP1d–CDP12d); the remaining five, which did not satisfy all criteria, were retained for reference as Pocket-13d to Pocket-17d (Table S4). Among the 12 CDPds, one localized exclusively to the MTase domain (CDP10d), six to the RdRp domain (CDP1d, CDP4d, CDP5d, CDP7d, CDP11d, CDP12d), and five spanned both domains (CDP2d, CDP3d, CDP6d, CDP8d, CDP9d); the full per-pocket assignment is provided in Table S4. As expected, dimer-associated CDPds were larger than their monomeric counterparts, an advantageous feature for ligand binding owing to the increased interaction surface.

**Figure 5:**
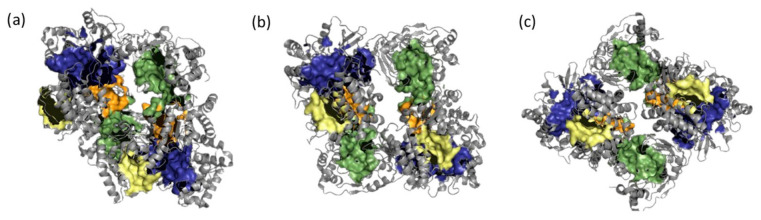
Structural mapping of the leading dimer Consensus Druggable Pockets (CDPds) candidates onto the DENV NS5 (PDB ID: 5ZQK). The four selected pockets are highlighted in distinct colors: CDP1d (green), CDP3d (blue), CDP5d (yellow), and CDP12d (orange). Multiple orientations (**a**–**c**) are shown to illustrate the spatial arrangement of these pockets within the dimer structure.

Four CDPds combine the most favourable features for antiviral targeting and were mapped onto the NS5 dimer structure (Figure 5). CDP1d, the largest pocket identified (68 residues), extends across the Fingers, Palm, and Thumb subdomains, including residues of the priming loop (residues 782–808) [18], qualifying it as a compelling target. CDP3d (57 residues) spans the MTase Core, linker, and RdRp Fingers subdomains, and encompasses all SAM (S-adenosyl-L-methionine)-binding residues (S56, K61, C82, G86, W87, T104, K105, D131, V132, F133, D146, I147, K181, E217), including the MTase catalytic tetrad (K61, D146, K181, E217), directly linking it to the methyltransferase catalytic core [29,32]. CDP5d (17 residues, RdRp Thumb) includes several residues of the priming loop. CDP12d (25 residues, RdRp Palm) covers nearly all residues of the polymerase catalytic site—the aspartates of motif A (D533, D534), the GDD catalytic triad of motif C (positions 662–664), and most residues of motif D (5/9; residues 681–689) [17,33]. Several additional pockets also overlap functionally important regions—including further SAM-interacting residues, the GTP-binding site, and conserved RdRp motifs A-E—as detailed in Table S4.

Together, these findings identify a subset of pockets within the NS5 dimer that combine large size, interdomain localization, and overlap with key functional elements, defining high-value potential targets for structure-based antiviral design. Dimer-associated CDPds emerge as particularly promising candidates for pharmacological modulation, given that the dimer conformation is believed to be the protein’s potentially active state. Furthermore, their larger size relative to monomeric RdRp CDPs, provides expanded interaction surfaces that may facilitate ligand binding and stabilization.

Among the four prioritized CDPds, CDP1d and CDP3d emerged as the top-ranked candidates, owing to their higher druggability scores, higher T-RHS content, and extended length. Specifically, CDP1d has a drug score of 0.84, contains 27 T-RHS, and spans 68 residues, while CDP3d has a drug score of 0.73, contains 36 T-RHS, and spans 57 residues. CDP12d is further distinguished by its extensive overlap with the monomer-associated CDP2 (the most promising pocket of the monomeric form) sharing 22 residues (Table S4). Structural superimposition of these shared residues confirmed their close correspondence (RMSD = 0.0 Å over the shared residues; Figure S3), highlighting this region as a conserved, structurally robust target across distinct NS5 conformations.

CDP1d, CDP5d, and CDP12d map exclusively to the RdRp domain, whereas CDP3d spans both domains with a predominance in the MTase. The RdRp domain harbours more numerous and larger CDPds, while the MTase domain shows a higher T-RHS prevalence (21.3% vs. 14.7%), consistent with its marginally higher conservation (Section 2.2). Although pocket number alone is not a determinant of therapeutic success, the RdRp domain remains the most promising target on biological grounds: it constitutes the catalytic core of viral RNA replication, and its structural elements are directly coupled to polymerase function and the replicative cycle in a way that the MTase domain is not. The MTase nonetheless offers densely conserved, functionally critical sites (most notably the SAM-binding and catalytic-tetrad residues captured by CDP3d) that justify retaining prioritized pockets from both domains for further functional investigation.

### 2.4. Bridging Identified Pockets with Putative NS5 Inhibitors

Despite the substantial number of preclinical candidates with reported activity against DENV NS5 (Table S1), only a limited subset has defined interaction sites, and these are largely derived from preliminary docking simulations rather than experimentally validated structures. One exception is RK-0404678, co-crystallized with NS5-RdRp, revealing two distinct binding sites: Site 1 at the Thumb–Palm interface, and Site 2 at the RNA tunnel adjacent to the catalytic center. Binding at these sites induces conformational rearrangements of the α16 helix and its flanking loop, ultimately disrupting the catalytic core [34]. Notably, Site 1 overlaps extensively with CDP5d identified here, sharing all but one residue (namely, S763, R773, N777, C780, D808, M809, W833, and Y882; Tables S1 and S4). Furthermore, recent docking studies indicate that the approved drugs doxorubicin and rifamycin bind to a similar region as RK-0404678, sharing four common interacting residues, and likewise overlapping with CDP5d (Table S1). Collectively, these findings reinforce CDP5d as a promising and functionally relevant targetable region within the NS5 dimer. 

Beyond CDP5d, several other pockets show considerable overlap with reported binding sites of preclinical anti-DENV NS5 candidates, further supporting the relevance of the identified CDPds and the robustness of the present findings. CDP3d and Pocket-16d encompass all or nearly all of the interacting residues identified for compound 10 (co-crystallized with NS5-MTase) and for the docking-derived compounds NSC 12155 and cordycepin—the latter representing the only natural product with characterized interaction profile (Tables S1 and S4). A significant proportion of the interacting residues of the two synthetic compounds—compound 10 and NSC 12155—are also contained within CDP8d and CDP9d. As for CDP5d, these observations indicate a convergence of structurally distinct ligands toward common regions that coincide with identified CDPds. 

The active metabolite of AT-752 (AT-9010) (co-crystallized in complex with NS5-MTase) exhibits 9 key interacting residues located within and around the GTP-binding site, most of which overlap with CDP10d, previously identified as coinciding with this functionally critical region (Section 2.3.2). Although AT-9010 is a nucleotide analogue, it has been shown to exhibit dual activity as both a chain terminator and a 2′-O-methyltransferase inhibitor, interacting with both domains of NS5 [35]. Notably, AT-752 is one of the few DENV inhibitors to have advanced to clinical trials; although these were discontinued without detailed justification [36]. Another well-known nucleotide analog, sofosbuvir—approved for the treatment of HCV infections and widely explored for repurposing—has been predicted by docking to bind the catalytic residues D533, D663 and D664, and additionally the residue S600 in DENV NS5, which largely map to CDP12d and Pocket-15d (Table S4) [37]. Finally, CDP1d, the largest pocket identified, emerges as a relevant interaction region for several preclinical candidates. The non-nucleoside inhibitor Q63 shares approximately half of its interacting residues with CDP1d and Pocket-15d; whereas tadalafil and galidesivir (BCX4430) exhibit broad interaction profiles (>30 residues), with partial overlap across CDP1d, CDP2d and additional pockets, depending on the compound. A comparable pattern is observed for remdesivir—currently approved for COVID-19 and widely explored for repurposing due to its broad antiviral potential [7]—which displays a broad interaction network with limited overlap with CDP1d, and progressively less with CDP3d and Pocket-15d (Tables S1 and S4).

Collectively, these analyses identify CDP1d-CDP3d, CDP5d, CDP8d-CDP10d, CDP12d, Pocket-15d and Pocket-16d—as recurrent binding hot spots engaged by structurally diverse NS5 ligands, reinforcing their functional relevance and supporting their suitability as promising targetable regions for antiviral intervention. Ultimately, these structural insights provide a robust framework for refining preclinical candidates engaging these pockets, enabling the optimization of ligand-protein interactions toward structurally robust regions with enhanced resilience to resistance. The convergence of chemically distinct, experimentally or computationally characterized inhibitors onto this specific subset of pockets—rather than onto arbitrary surface regions—provides retrospective support for the predictive value of our consensus druggability approach. Of note, the engagement of Pocket-15d by several inhibitors (e.g., sofosbuvir, Q63), despite its sub-threshold druggability score, indicates that predicted druggability and observed ligand occupancy need not fully coincide; such regions may still warrant consideration as auxiliary interaction sites. Conversely, four candidate pockets—CDP4d, CDP6d, CDP7d, and CDP11d—showed no overlap with any reported inhibitor, representing conserved, previously unexploited druggable regions that constitute novel candidate targets arising from this work and prime candidates for prospective ligand discovery.

To assess the identified pockets as small-molecule binding sites, a preliminary docking analysis was performed against the four most promising CDPds: CDP1d, CDP3d, CDP5d, and CDP12d, using representative preclinical candidates, including non-nucleoside allosteric inhibitors (Q63, NSC 12155, and 25-deacetyl-rifamycin—the active form of rifamycin) and the nucleotide analogue remdesivir (GS-441524). All candidates displayed strong binding to the CDPs with which they share interacting residues, as evidenced by binding affinities below the defined threshold (−8.1 kcal/mol). Notably, NSC 12155 and 25-deacetyl-rifamycin also bound strongly to additional pockets (Table 3). These binding-affinity values should be interpreted in relative rather than absolute terms: docking scores scale with ligand size, as larger compounds establish a greater number of protein contacts and consequently yield more negative values irrespective of binding specificity. This is evident for 25-deacetyl-rifamycin, a large ligand that bound all four pockets with uniformly high affinities (−13.0 to −13.9 kcal/mol), a pattern reflecting its extensive contact surface rather than selective engagement of any single site. Accordingly, the relevant readout of this analysis is not the absolute affinity but the concordance between the predicted binding poses and the literature-reported interacting residues for each compound (indicated in Table S1), which consistently mapped to the pockets with which each ligand shares those residues.

**Figure 6:**
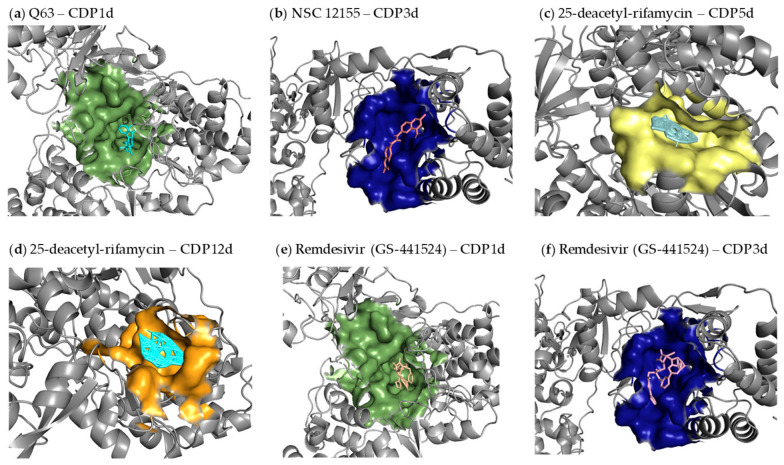
Structural visualization of representative preclinical candidates bound to leading CDPds with binding affinities below the defined threshold. Selected candidates include Q63 (**a**), NSC 12155 (**b**), 25-deacetyl-rifamycin (**c**,**d**), and remdesivir (GS-441524) (**e**,**f**). Pockets are colored as follows: CDP1d (green), CDP3d (blue), CDP5d (yellow), and CDP12d (orange).

Overall, all leading CDPs were bound by at least one compound, supporting their ability to accommodate small molecules and serve as viable interaction sites. Representative docking poses of these compounds within the distinct CDPds are shown in Figure 6, providing structural insights into ligand accommodation within these pockets. Detailed molecular interactions are presented in Figure S4. While these computational results do not establish physiological binding affinity, they provide structural evidence that the prioritized pockets can accommodate known NS5 ligands at their expected sites.

By leveraging this target-specific information to optimize existing molecules or guide the design of new ones, researchers can aim to enhance their binding affinity and inhibitory potency, toward structurally robust, resistance-resilient regions of DENV NS5.

## 3. Materials and Methods

### 3.1. Dataset Construction and Sequence Analysis

All complete genome-wide nucleotide sequences of globally circulating DENV were retrieved on 10 October 2023 from the NCBI Virus database (https://www.ncbi.nlm.nih.gov/labs/virus/vssi/#/ (accessed on 15 January 2024)) [38]. A total of 4395 nucleotide sequences were collected, including 1843 DENV1, 1460 DENV2, 859 DENV3 and 233 DENV4 sequences. Non-complete and duplicated genome sequences were removed, resulting in a final whole-genome DENV dataset of 4127 sequences (1707 DENV1, 1371 DENV2, 831 DENV3 and 218 DENV4). NS5 coding regions were then extracted according to genome coordinates to generate the corresponding protein sequence datasets for downstream analyses. Sequence alignments were performed by Clustal Omega or Multiple Alignment Fast Fourier Transform (MAFFT) methods, using the Job Dispatcher EMBL’s European Bioinformatics Institute online platform, available at https://www.ebi.ac.uk/jdispatcher/msa (accessed on 15 January 2024) [39]. Multiple sequence alignments were visualized, and protein sequence features, including primary structure and length variations across and within DENV serotypes, were analyzed using MEGA 11 [40]. Amino acid positions are referenced according to DENV2 numbering.

### 3.2. Conservation Analysis 

The conservation at each aa position in DENV NS5 was calculated using the Valdar scoring method via the Jalview AACons Web Server (version 2.11.3.2), as previously described by our team [41,42,43]. This scoring method has the key advantage of enabling the normalization against sequence redundancy and bias without losing evolutionary information [44]. Conservation scores are presented on a numerical scale, ranging from 0 (highly variable site) to 11 (highly conserved site). Residues with a conservation score of 11 correspond to absolute conserved sites (100% aa identity). Residues were classified as highly conserved (score of 10), conserved (score of 7–9), variable (score of 4–6), and highly variable (score ≤ 3). Conservation scores were calculated globally across DENV serotypes and separately for each serotype.

### 3.3. Druggability Analysis

Druggability studies were performed using multiple crystallographic structures of DENV NS5 available from the RCSB Protein Data Bank (https://www.rcsb.org) (accessed on 25 April 2024) [45]. Four crystallographic structures were selected (as depicted in Table S5): two structures containing the RdRp domain in a monomer conformation (PDB entries 6J00 and 6IZY); and two full-length NS5 structures encompassing both MTase and RdRp domains in a dimer conformation (PDB entries 5CCV and 5ZQK). Every structure was accurately prepared using the Molecular Operating Environment (MOE) software, version 2020.0901 (Chemical Computing Group, Montreal, QC, Canada). The druggable pockets and residues within each selected structure were predicted from a consensus dual-based strategy, using the webserver DoGSiteScorer (DGSS) (https://proteins.plus) (accessed on 10 July 2024) [46] and the SiteFinder tool in MOE software (MOE-SF), as previously described by our team [41,42,43]. The DGSS server is able to predict potential druggable pockets on the protein surface based only on geometric (size and shape) and physicochemical descriptors, using a grid-based method, while MOE-SF uses a geometry-based method that considers receptor size, number of contacts with the receptor, and chemical property information to predict potential pockets for drug targeting. Based on the premise that a minimum pocket size is necessary to allow a proper interaction between the target and putative ligands, only pockets with a minimum of 10 aa and with a druggability score > 0.4 (on a scale between 0 and 1) were taken into consideration. The top ranked sites with a positive score were considered druggable. An extended analysis was performed to merge the druggability information from both bioinformatic tools for each group: NS5-RdRp monomer and NS5 dimer conformations.

The last stage of the study consisted of a global comparative analysis of the most prevailing druggable sites identified by the two bioinformatics tools and shared by the structures within each group. Druggable pockets predicted independently by MOE-SF and DGSS were compared and only those identified by both tools were retained as consensus pockets; pockets predicted by a single tool were not considered. Pockets were merged into a single Consensus Druggable Pocket (CDP), when they shared all constituent residues and their spatial proximity was confirmed by visual inspection of the three-dimensional structure in MOE and PyMOL Molecular Graphics System, version 3.1.0 (Schrödinger, LLC, New York, USA). In ambiguous cases, structural superimposition was used to confirm correspondence between pockets using PyMOL. The resulting druggability consensus of each group was then analysed in parallel with conservation data to identify the CDPs and the potential top-ranked hot spot (T-RHS) residues (i.e., residues with the highest combined scores for conservation and druggability). Structural visualization and final mapping of CDPs onto the 5ZQK structure were performed using PyMOL.

### 3.4. Molecular Docking

Representative preclinical candidates were retrieved from distinct databases: NSC 12155 from Pubchem (ID: 79472) [47]; and 25-deacetyl-rifamycin (ID: DBMET02189) and remdesivir (ID: GS-441524) from DrugBank [48]. Q63 was exceptionally drawn using the ChemTradeHub Chemical Structure Editor online tool (https://www.chemtradehub.com/tools/structure-editor/P) (accessed on 20 May 2026). Docking was performed using AutoDock Vina, version 1.1.2 [49], against the leading CDPds (CDP1d, CDP3d, CDP5d, and CDP12d) mapped onto the DENV2 NS5 structure (PDB ID: 5ZQK). Binding affinities and top-ranked poses were recorded for each compound–pocket pair. A binding threshold of −8.1 kcal/mol was defined based on docking of crystallographic ligands at their native binding sites—specifically, SAM in the 5ZQK structure (MTase domain) and 68E in the 5JJR structure (RdRp domain). Values equal or more negative than this threshold were considered indicative of strong binding. Docking poses were visualized in PyMOL and ligand–protein interactions were analyzed using BIOVIA Discovery Studio Visualizer, version 25.1.0 (Dassault Systèmes, Vélizy-Villacoublay, France).

## 4. Conclusions

To the best of our knowledge, this study represents the first comprehensive analysis integrating and comparing the conservation and druggability of DENV NS5 protein. Our approach leverages an extensive set of sequences that provide a representative depiction of the global diversity and circulation patterns of the different DENV serotypes, together with the most robust and well-characterized NS5 conformations available, encompassing both the RdRp monomer and the full-length NS5 dimer. Crucially, whereas the large existing portfolio of NS5-targeting candidates has been developed largely without structure-based design and without a comprehensive assessment of sequence conservation (as detailed in Introduction), our framework prioritizes targets on both criteria simultaneously–identifying conserved druggable regions that previous, conservation-agnostic efforts could not systematically pinpoint.

Our findings show that, regardless of the NS5-RdRp conformational state, conserved regions across DENV serotypes can overlap with potential binding sites, supporting the potential of DENV NS5 as a valuable antiviral target with an anticipated high barrier to resistance. Notably, while the RdRp domain exhibits a greater number and broader druggable surfaces, the MTase domain is enriched in conserved regions that are often functionally and/or structurally critical, underscoring the value of prioritizing both domains for antiviral intervention. The RdRp domain nonetheless remains the foremost target on biological grounds, as it constitutes the catalytic core of viral RNA replication and its structural elements are directly coupled to polymerase function and the replicative cycle. Large druggable pockets are highly desirable, as they provide greater structural capacity for ligand accommodation, facilitating stronger and more specific interactions (binding affinity and specificity). Targeting highly conserved regions offers a further, dual advantage: because such sites are evolutionarily constrained, compounds directed against them are expected to face a higher genetic barrier to resistance and to retain activity across serotypes.

The identification of CDP1d, CDP3d, CDP5d, and CDP12d as promising antiviral targets enables, for the first time, the directed search for novel anti-DENV agents toward robust, structurally based targets with an expected resilience to resistance and pan-serotype potential. In parallel, the structural insights provided by our CDPds offer a robust platform for refining known antiviral compounds targeting DENV NS5, particularly those binding to regions overlapping the identified CDPds. This knowledge is crucial for guiding chemical optimization efforts, facilitating the elucidation of their precise mechanisms of action, and ultimately enhancing their potency against DENV.

As with any in silico approach, the regions and interactions identified here are computational predictions that define favourable structural and evolutionary features rather than confirmed binding events, and therefore represent a starting point for experimental investigation. Two complementary strategies can be pursued to this end. First, comprehensive virtual screening and molecular docking can evaluate the binding affinity of diverse ligands within the most promising CDPds, providing a rational route for the discovery of novel candidate compounds, an avenue in which drug repurposing also holds significant promise. Direct engagement of the prioritized pockets (CDP1d, CDP3d, CDP5d, CDP12d) can then be confirmed using recombinant full-length NS5 and its isolated RdRp/MTase domains through biophysical assays (e.g., surface plasmon resonance, microscale thermophoresis, or isothermal titration calorimetry), and the functional consequence of binding assessed through domain-specific RdRp polymerase and MTase methyltransferase assays. The efficacy of candidate compounds should ultimately be confirmed through in vitro and in vivo assays, yielding essential data on antiviral potency, specificity, toxicity, and resistance profiles. Second, mutagenesis of selected residues within the prioritized CDPds can assess their functional importance in viral replication, providing mechanistic validation of the identified pockets and a direct experimental test of the resistance barrier that our conservation-based rationale predicts. We note that the genetic barrier to resistance of a given target is, in practice, assessed once inhibitors with confirmed antiviral activity against that target are available—either through resistance-selection assays on defined-target compounds or, for compounds of unknown mechanism, through the emergence of resistant strains used to map the target itself. Accordingly, experimental determination of the resistance barrier for the regions prioritized here represents a subsequent stage, building on the conservation-based rationale established in this work. Together, this integrated approach of ligand discovery, optimization, and experimental validation is expected to accelerate the development of broad-spectrum, resistance-resilient DAAs against DENV.

Scientific progress in this area must be contextualized within a One Health framework. The ongoing geographic expansion of DENV into industrialized countries—a tangible result of climate change on vector migration—highlights an urgent need for novel antiviral solutions and greater investment in dengue research. This convergence of environmental and global health pressures creates a critical opportunity to prioritize and accelerate the development of effective and resistance-resilient antiviral options against DENV. 

By enabling the identification of conserved and potentially druggable pockets within DENV NS5, this study provides a powerful framework for the rational design of DAAs and addresses a longstanding gap in the field: the historical focus on structural proteins. A paradigm shift toward targeting NS proteins is warranted by their high conservation across serotypes, which is expected to confer a broader spectrum of activity and a lower likelihood of resistance. Finally, our findings hold implications beyond DENV. Current evidence supports that the NS5 protein exhibits a high level of conservation not only across DENV serotypes but also among other flaviviruses such as Zika and Japanese encephalitis virus [28]. These findings raise the possibility that the present framework could support the development of broad-spectrum antiviral strategies against multiple flaviviral threats.

## Figures and Tables

**Table 1:** Distribution of DENV NS5 protein lengths across serotypes and their specific features.

	NS5 Length Type (LT)								Specific Features				
DENV Serotype	896	897	898	899		900	901	Total	NS5 LT		Amino Acid Profile	Protein Domain	Temporal and Spatial Distribution of Viruses
DENV1 (1944–2023)	1	1	1	1704				1707 (41.4%)	896		del aa 100–102	Mtase (Core subdomain)	2007, Yucatan (Mexico)
									897		del aa 338 + 346	RdRp	2007, Vietnam
									898		del aa 346	RdRp	2007, Vietnam
									899		–	–	1944–2023, Worldwide
DENV2 (1944–2023)				2		1369		1371 (33.2%)	899	LT1	del aa 176	Mtase (Core subdomain)	2015, Australia
				LT1	LT2					LT2	del aa 282	RdRp (Fingers subdomain)	2009, California (USA)
				1	1				900		–	–	1944–2023, Worldwide
DENV3 (1956–2023)				1		829	1	831 (20.1%)	899		del aa 389	RdRp	2009, Nicaragua
									900		–	–	1944–2023, Worldwide
									901		ins aa 612–613	RdRp (Palm subdomain)	2002, Puerto Rico (USA)
DENV4 (1953–2021)						218		218 (5.3%)	900		**–**	**–**	1944–2023, Worldwide
TOTAL	1 (0.00024%)	1 (0.00024%)	1 (0.00024%)	1707 (41.4%)		2416 (58.5%)	1 (0.00024%)	4 127					

del: deletion; inser: insertion; aa: amino acid; MTase: Methyltransferase; RdRp: RNA-dependent RNA Polymerase. Length types (LTs) with a prevalence greater than 1% are underlined, and the predominant LT for each serotype is highlighted in blue.

**Table 2:** Percentage of NS5 residues assigned to each conservation category by DENV serotype.

	DENV1	DENV2	DENV3	DENV4
% highly conserved residues	96.6	94.1	98.9	96.2
% conserved residues	2.6	3.2	1.0	3.0
% variable residues	0.4	1.3	–	0.6
% highly variable residues	0.4	1.3	0.1	0.2

**Table 3:** Binding affinities (kcal/mol) of selected preclinical candidates with reported anti-DENV NS5 activity, evaluated against the leading target candidates CDP1d, CDP3d, CDP5d, and CDP12d.

Compound	CDP1d	CDP3d	CDP5d	CDP12d
Q63	−8.8 *	−7.8	−6.0	−6.9
NSC 12155	**−8.4**	−8.5 *	−7.8	−8.0
25-deacetyl-rifamycin	**−13.9**	**−13.7**	−13.2 *	**−13.0**
Remdesivir (GS-441524)	−10.9 *	−10.8 *	−7.0	−7.6

A binding threshold of −8.1 kcal/mol was defined based on docking of crystallographic ligands at their native binding sites: SAM in the 5ZQK structure (MTase domain); and 68E in the 5JJR structure (RdRp domain). Values below this threshold (i.e., more negative values) indicative of strong binding are shown in bold. The asterisk (*) indicates overlap between literature-reported interacting residues and CDPd residues.

## Data Availability

The sequence datasets constructed and analyzed in this study are available on request from the corresponding author.

## Supplementary Materials

The following supporting information can be downloaded at: https://www.mdpi.com/article/10.3390/ijms27125639/s1.

## Abbreviations

The following abbreviations are used in this manuscript:

CDP	Consensus Druggable Pocket
CDPd	Dimer’s Consensus Druggable Pocket
DAA	Direct-Acting Antiviral
DENV	Dengue virus
DGSS	DoGSiteScorer
E	Envelope
LV	Length Variant
MTase	Methyltransferase
NS	Non-Structural
NS5	Non-structural protein 5
RdRp	RNA-dependent RNA Polymerase
