# Supplementary material for: Dengue Virus NS5 Target Discovery: A Comprehensive in Silico Exploration of Novel Druggable Sites for Pan-Serotype Antiviral Design
Source: Int J Mol Sci. 2026 Jun 22;27(12):5639. doi: 10.3390/ijms27125639 (PMC13299206; doi:10.3390/ijms27125639)
Supplement: Supplementary file 1 [file ijms-27-05639-s001.zip › Figure_S1.pdf]

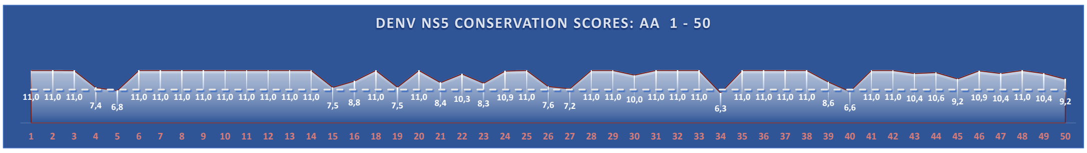
DENV NS5 CONSERVATION SCORES: AA 1 - 50

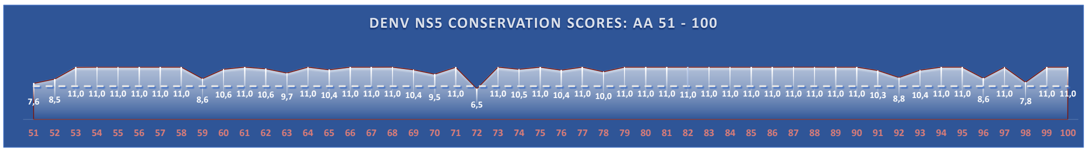
DENV NS5 CONSERVATION SCORES: AA 51 - 100

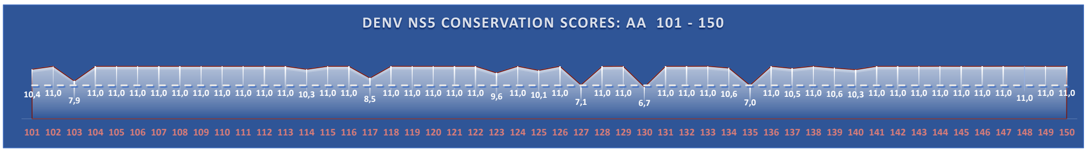
DENV NS5 CONSERVATION SCORES: AA 101 - 150

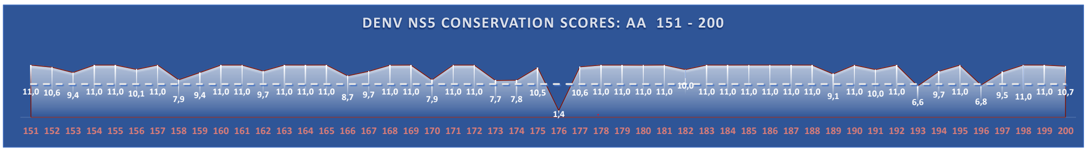
DENV NS5 CONSERVATION SCORES: AA 151 - 200

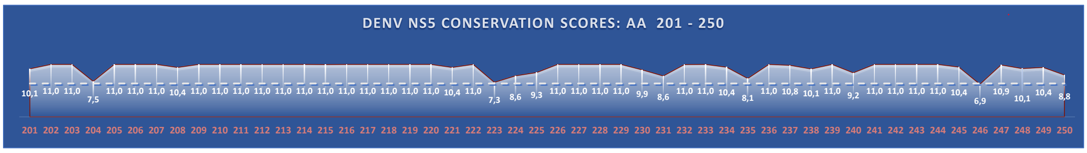
DENV NS5 CONSERVATION SCORES: AA 201 - 250

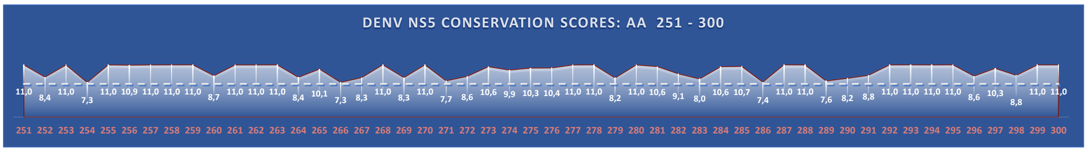
DENV NS5 CONSERVATION SCORES: AA 251 - 300

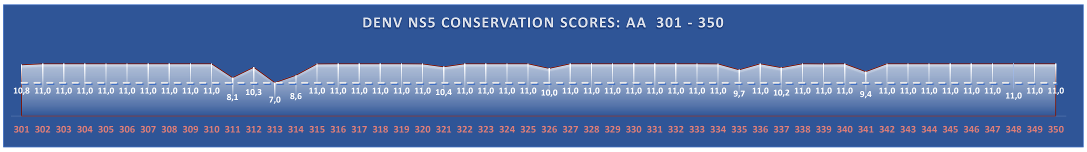
DENV NS5 CONSERVATION SCORES: AA 301 - 350

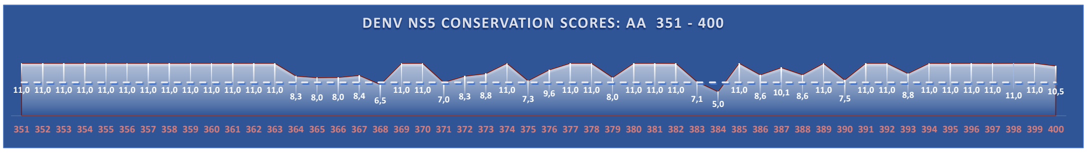
DENV NS5 CONSERVATION SCORES: AA 351 - 400

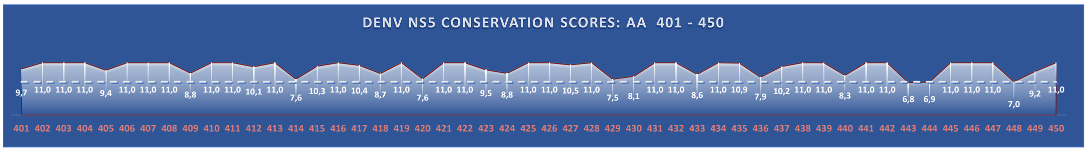
DENV NS5 CONSERVATION SCORES: AA 401 - 450

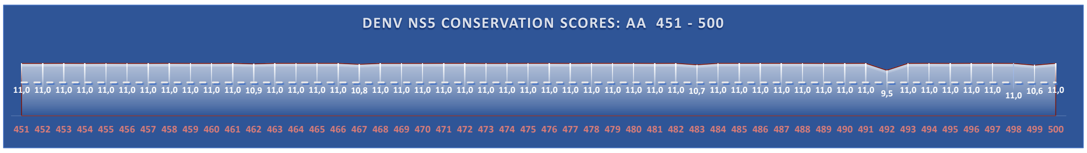
DENV NS5 CONSERVATION SCORES: AA 451 - 500

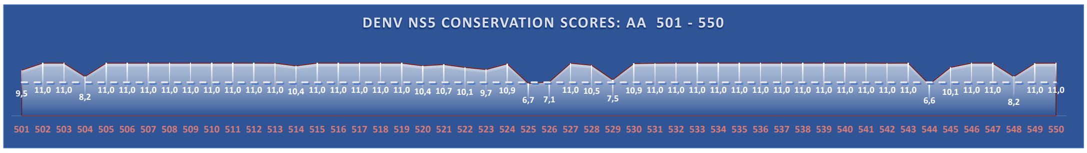
DENV NS5 CONSERVATION SCORES: AA 501 - 550

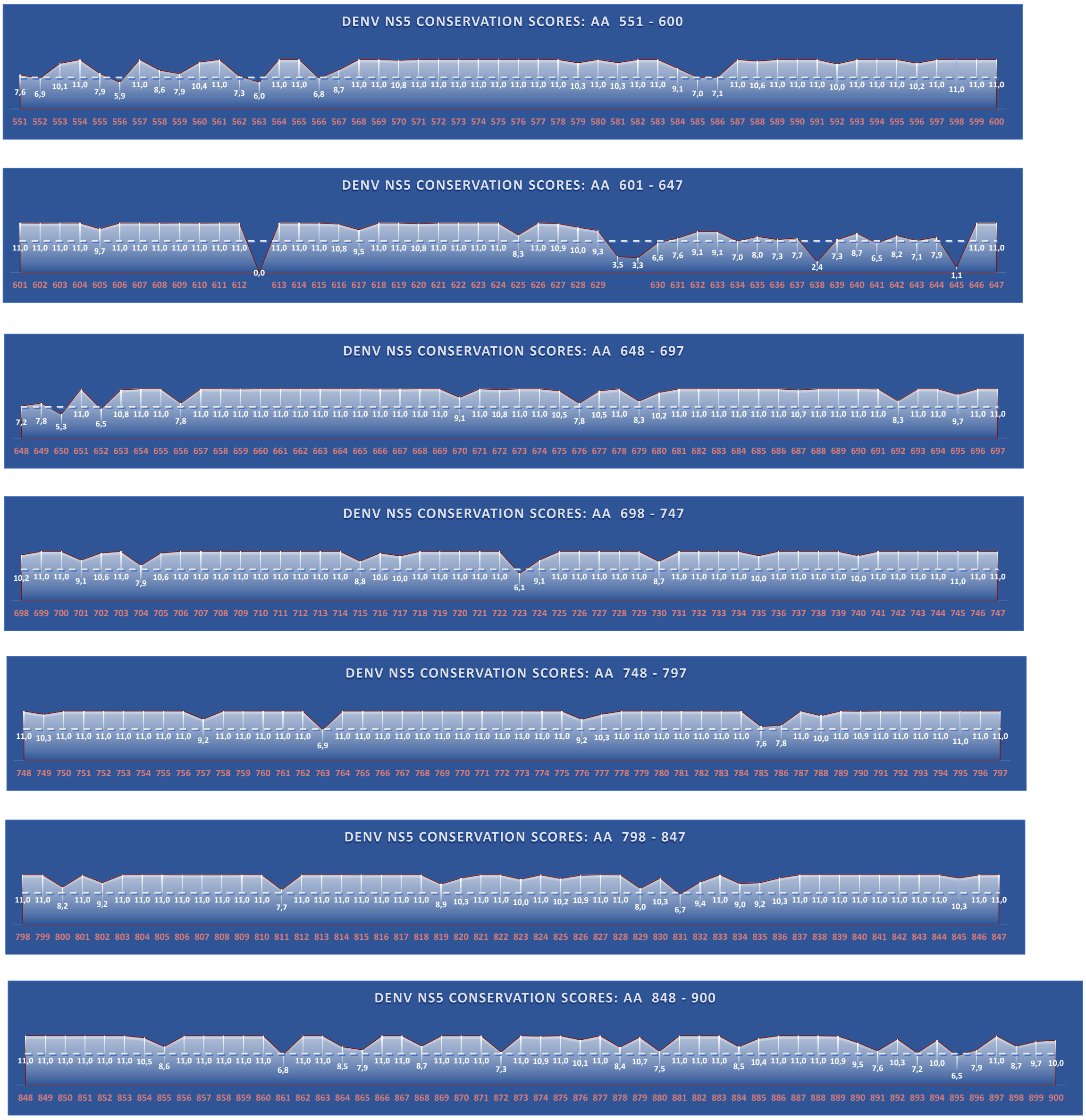

Figure S1. Distribution of the global conservation scores along the DENV NS5 protein. The numbers below each site represent the conservation score of each individual residue, on a scale varying from 0 (most variable) to 11 (most conserved), with a score of 11,0 indicating 100% conservation across the four serotypes (DENV1 to DENV4). Residue numbering is based on DENV2 coordinates.
