# Supplementary material for: Dengue Virus NS5 Target Discovery: A Comprehensive in Silico Exploration of Novel Druggable Sites for Pan-Serotype Antiviral Design
Source: Int J Mol Sci. 2026 Jun 22;27(12):5639. doi: 10.3390/ijms27125639 (PMC13299206; doi:10.3390/ijms27125639)
Supplement: Supplementary file 1 [file ijms-27-05639-s001.zip › Figure_S2.pdf]

aa	1	2	3	4	5	6	7	8	9	10	11	12	13	14	15	16	17	18	19	20	21	22	23	24	25	26	27	28	29	30	31	32	33	34	35	36	37	38	39	40	41	42	43	44	45	46	47	48	49	50
DENV-1	10,96	10,96	10,89	11,00	10,99	11,00	11,00	11,00	11,00	11,00	11,00	11,00	11,00	10,99	11,00	10,79	11,00	10,99	11,00	11,00	10,95	11,00	10,49	11,00	11,00	10,96	8,06	11,00	10,99	10,79	11,00	11,00	11,00	10,59	11,00	11,00	10,97	11,00	10,92	11,00	10,98	10,99	11,00	11,00	10,95	10,77	11,00	11,00	11,00	10,73
DENV-2	10,97	11,00	10,90	10,76	1,70	10,92	10,95	10,97	10,96	10,92	10,96	10,99	10,97	11,00	3,11	10,91	10,85	10,85	9,05	10,93	10,98	10,98	10,70	10,97	10,96	10,95	9,93	11,00	11,00	10,28	11,00	10,97	10,95	10,92	11,00	11,00	11,00	11,00	10,95	10,95	11,00	10,96	10,97	11,00	10,98	10,78	10,96	10,97	10,97	11,00
DENV-3	11,00	11,00	11,00	10,93	10,99	11,00	11,00	11,00	11,00	10,99	11,00	11,00	11,00	11,00	10,92	10,97	11,00	11,00	11,00	11,00	10,99	11,00	10,97	10,45	11,00	10,97	10,96	10,98	11,00	10,97	11,00	11,00	11,00	11,00	11,00	11,00	10,99	11,00	10,94	10,99	11,00	11,00	10,99	11,00	11,00	10,99	10,98	11,00	11,00	8,94
DENV-4	10,83	11,00	11,00	10,74	6,87	11,00	11,00	11,00	11,00	11,00	11,00	10,86	10,83	11,00	11,00	10,89	11,00	11,00	11,00	11,00	10,85	11,00	10,90	10,83	11,00	11,00	11,00	11,00	11,00	10,95	11,00	11,00	11,00	10,58	11,00	11,00	11,00	11,00	10,81	11,00	10,84	10,58	10,64	10,77	11,00	9,24	10,85	11,00	9,93	10,95

aa	51	52	53	54	55	56	57	58	59	60	61	62	63	64	65	66	67	68	69	70	71	72	73	74	75	76	77	78	79	80	81	82	83	84	85	86	87	88	89	90	91	92	93	94	95	96	97	98	99	100
DENV-1	10,96	9,98	11,00	10,98	11,00	11,00	11,00	11,00	10,89	11,00	11,00	11,00	10,99	11,00	10,99	11,00	10,99	11,00	10,95	10,97	11,00	10,91	11,00	11,00	11,00	10,99	11,00	10,97	10,98	11,00	11,00	10,98	11,00	11,00	10,97	10,96	11,00	11,00	10,99	11,00	11,00	11,00	11,00	11,00	10,99	10,97	10,95	10,98	11,00	10,96
DENV-2	10,95	10,98	11,00	11,00	11,00	10,89	10,89	10,91	10,94	11,00	11,00	11,00	11,00	10,97	11,00	11,00	11,00	11,00	10,97	6,33	11,00	10,85	11,00	10,97	10,96	10,95	10,96	10,05	11,00	11,00	10,97	11,00	10,97	11,00	11,00	11,00	11,00	11,00	10,96	11,00	11,00	10,98	11,00	11,00	10,98	10,46	11,00	8,20	10,98	11,00
DENV-3	10,96	10,93	11,00	11,00	11,00	11,00	11,00	11,00	10,86	11,00	11,00	11,00	11,00	11,00	10,98	11,00	11,00	11,00	10,98	11,00	10,99	10,92	11,00	11,00	11,00	11,00	10,99	10,99	10,98	10,99	10,99	11,00	11,00	10,98	11,00	11,00	11,00	11,00	11,00	11,00	11,00	11,00	11,00	11,00	11,00	11,00	11,00	11,00	11,00	11,00
DENV-4	8,04	10,70	9,25	10,86	10,89	11,00	10,81	11,00	7,53	10,81	11,00	11,00	11,00	10,78	11,00	10,63	11,00	11,00	10,67	11,00	9,26	10,89	11,00	10,95	11,00	10,94	10,81	10,56	10,84	10,78	11,00	11,00	11,00	11,00	11,00	11,00	11,00	11,00	10,95	11,00	10,84	11,00	11,00	10,81	10,75	10,72	11,00	10,88	11,00	11,00

aa	101	102	103	104	105	106	107	108	109	110	111	112	113	114	115	116	117	118	119	120	121	122	123	124	125	126	127	128	129	130	131	132	133	134	135	136	137	138	139	140	141	142	143	144	145	146	147	148	149	150
DENV-1	10,95	10,96	10,97	10,99	10,95	10,98	10,97	10,85	10,96	10,92	10,95	10,92	10,98	10,77	10,96	10,98	10,96	11,00	10,97	10,98	10,98	10,95	9,26	10,94	10,97	10,95	10,19	10,98	11,00	10,07	10,99	10,88	11,00	11,00	3,09	10,98	10,87	11,00	10,97	10,97	10,98	10,98	10,99	11,00	10,97	11,00	11,00	11,00	11,00	11,00
DENV-2	10,99	11,00	10,97	11,00	10,98	11,00	11,00	11,00	11,00	11,00	11,00	11,00	11,00	10,94	10,95	11,00	10,98	11,00	11,00	11,00	11,00	11,00	10,95	10,98	10,99	11,00	10,78	10,88	11,00	10,65	11,00	11,00	11,00	10,99	5,53	10,87	10,92	11,00	10,93	11,00	10,98	11,00	10,98	11,00	10,97	11,00	11,00	11,00	10,96	10,97
DENV-3	11,00	11,00	11,00	11,00	11,00	10,99	11,00	11,00	11,00	11,00	11,00	10,99	11,00	10,98	11,00	11,00	10,96	11,00	11,00	11,00	11,00	11,00	10,77	10,98	11,00	11,00	10,99	10,92	11,00	11,00	11,00	11,00	11,00	11,00	11,00	10,99	11,00	11,00	10,98	11,00	11,00	11,00	11,00	11,00	11,00	10,99	11,00	11,00	11,00	11,00
DENV-4	10,84	11,00	10,98	11,00	11,00	11,00	11,00	11,00	11,00	11,00	11,00	11,00	11,00	11,00	11,00	11,00	11,00	11,00	11,00	11,00	11,00	11,00	11,00	10,86	11,00	11,00	11,00	10,88	10,84	11,00	10,89	11,00	11,00	11,00	10,83	11,00	10,74	11,00	11,00	11,00	11,00	11,00	11,00	11,00	11,00	11,00	11,00	11,00	11,00	10,88

aa	151	152	153	154	155	156	157	158	159	160	161	162	163	164	165	166	167	168	169	170	171	172	173	174	175	176	177	178	179	180	181	182	183	184	185	186	187	188	189	190	191	192	193	194	195	196	197	198	199	200
DENV-1	11,00	10,98	10,96	11,00	11,00	10,97	11,00	11,00	11,00	10,99	11,00	10,99	11,00	10,95	11,00	10,95	11,00	10,91	11,00	11,00	11,00	11,00	10,74	10,87	10,97		11,00	11,00	11,00	10,99	11,00	10,82	11,00	11,00	11,00	11,00	11,00	10,98	10,95	10,97	10,96	11,00	10,52	11,00	10,99	10,51	11,00	11,00	11,00	10,84
DENV-2	11,00	11,00	10,97	10,97	11,00	7,51	11,00	11,00	11,00	11,00	11,00	11,00	11,00	10,99	11,00	9,91	10,97	10,92	11,00	10,95	10,97	11,00	10,84	10,82	10,00	10,88	10,94	10,97	11,00	10,50	11,00	11,00	11,00	10,95	11,00	11,00	11,00	10,97	10,97	10,99	11,00	11,00	10,87	11,00	10,97	4,27	10,95	11,00	11,00	9,04
DENV-3	10,99	11,00	11,00	11,00	10,94	11,00	11,00	11,00	10,99	11,00	11,00	10,99	11,00	11,00	11,00	10,99	10,99	10,98	11,00	10,99	10,98	10,98	10,76	10,94	10,99		10,98	10,97	10,99	11,00	11,00	10,95	11,00	11,00	11,00	11,00	11,00	10,99	10,22	10,98	10,95	11,00	11,00	11,00	11,00	10,98	11,00	11,00	11,00	11,00
DENV-4	11,00	11,00	11,00	10,86	11,00	11,00	11,00	10,73	10,78	10,56	10,86	10,83	11,00	10,81	11,00	10,81	10,78	10,66	11,00	10,72	11,00	11,00	10,88	10,83	11,00	11,00	11,00	11,00	11,00	11,00	11,00	10,89	11,00	11,00	11,00	11,00	11,00	11,00	10,88	10,90	11,00	11,00	11,00	11,00	11,00	10,95	11,00	11,00	11,00	8,82

aa	201	202	203	204	205	206	207	208	209	210	211	212	213	214	215	216	217	218	219	220	221	222	223	224	225	226	227	228	229	230	231	232	233	234	235	236	237	238	239	240	241	242	243	244	245	246	247	248	249	250
DENV-1	10,64	11,00	11,00	11,00	11,00	10,99	11,00	11,00	11,00	11,00	10,97	11,00	11,00	11,00	10,93	11,00	11,00	11,00	10,97	11,00	10,99	11,00	10,96	10,98	11,00	11,00	11,00	11,00	11,00	11,00	11,00	10,79	11,00	11,00	11,00	11,00	11,00	11,00	11,00	11,00	11,00	11,00	10,99	11,00	10,95	10,46	11,00	11,00	10,99	11,00
DENV-2	10,78	11,00	11,00	10,94	10,94	11,00	11,00	11,00	11,00	10,97	11,00	10,97	10,96	10,96	10,96	10,97	11,00	11,00	11,00	11,00	11,00	11,00	11,00	11,00	2,29	11,00	11,00	10,98	11,00	10,97	10,97	11,00	11,00	11,00	11,00	11,00	11,00	10,96	11,00	10,99	11,00	11,00	10,98	11,00	11,00	7,48	10,50	11,00	10,96	10,87
DENV-3	10,50	11,00	11,00	10,99	11,00	11,00	11,00	10,97	11,00	11,00	11,00	11,00	11,00	11,00	10,98	10,97	11,00	11,00	10,96	11,00	11,00	11,00	10,99	11,00	10,95	11,00	11,00	11,00	10,99	8,77	10,98	11,00	10,99	10,88	10,99	11,00	11,00	11,00	11,00	11,00	11,00	11,00	10,97	10,95	11,00	10,95	10,80	11,00	11,00	10,99
DENV-4	10,00	11,00	11,00	4,27	11,00	10,95	11,00	11,00	11,00	11,00	11,00	11,00	11,00	11,00	11,00	11,00	11,00	11,00	11,00	11,00	11,00	11,00	10,86	6,78	10,38	10,47	10,75	10,90	11,00	11,00	11,00	11,00	11,00	11,00	7,45	11,00	11,00	11,00	11,00	11,00	11,00	11,00	11,00	11,00	10,72	10,46	10,28	11,00	11,00	11,00

aa	251	252	253	254	255	256	257	258	259	260	261	262	263	264	265	266	267	268	269	270	271	272	273	274	275	276	277	278	279	280	281	282	283	284	285	286	287	288	289	290	291	292	293	294	295	296	297	298	299	300
DENV-1	10,98	10,89	10,99	10,76	11,00	11,00	11,00	11,00	10,98	10,00	11,00	10,98	11,00	10,98	10,97	10,84	10,97	10,99	11,00	11,00	9,79	10,93	11,00	10,94	10,89	10,96	10,98	11,00	11,00	10,99	11,00	11,00	10,98	10,98	10,99	3,64	10,98	11,00	10,97	10,94	11,00	11,00	10,99	10,97	11,00	11,00	11,00	11,00	11,00	11,00
DENV-2	11,00	11,00	10,93	10,88	11,00	11,00	10,92	11,00	11,00	11,00	10,94	10,95	11,00	11,00	10,97	10,96	10,79	11,00	8,31	11,00	2,86	11,00	10,82	10,99	10,91	10,86	10,82	10,97	11,00	11,00	10,99	10,87	10,95	10,79	10,94	10,44	11,00	10,88	10,85	8,35	10,98	10,85	10,97	10,95	11,00	10,96	10,99	10,99	10,95	11,00
DENV-3	10,95	10,89	11,00	10,97	11,00	11,00	10,98	11,00	11,00	11,00	11,00	10,99	11,00	11,00	11,00	10,99	10,96	11,00	11,00	11,00	10,91	11,00	10,99	11,00	10,93	10,98	11,00	11,00	11,00	11,00	11,00	10,66	10,99	10,98	10,97	11,00	11,00	11,00	9,06	10,98	10,99	11,00	11,00	10,99	10,98	10,94	11,00	11,00	11,00	11,00
DENV-4	10,86	11,00	11,00	10,73	11,00	7,51	10,89	11,00	11,00	11,00	11,00	11,00	11,00	11,00	11,00	11,00	10,73	11,00	7,68	11,00	11,00	11,00	10,90	10,93	9,22	10,62	11,00	11,00	10,64	10,78	10,63	10,75	11,00	11,00	7,88	11,00	11,00	11,00	10,73	10,95	11,00	11,00	11,00	11,00	11,00	9,03	11,00	11,00	11,00	11,00

aa	301	302	303	304	305	306	307	308	309	310	311	312	313	314	315	316	317	318	319	320	321	322	323	324	325	326	327	328	329	330	331	332	333	334	335	336	337	338	339	340	341	342	343	344	345	346	347	348	349	350
DENV-1	10,99	11,00	11,00	11,00	11,00	11,00	11,00	10,98	11,00	10,99	10,92	10,96	10,95	11,00	10,98	10,96	10,98	11,00	11,00	11,00	10,97	11,00	11,00	10,97	11,00	8,26	11,00	11,00	11,00	11,00	11,00	11,00	11,00	10,94	11,00	10,98	10,98	10,96	10,96	10,98	10,90	11,00	11,00	11,00	10,99	10,92	10,97	10,96	10,95	10,96
DENV-2	10,76	10,97	10,97	11,00	10,96	10,99	11,00	11,00	11,00	11,00	10,95	10,96	11,00	11,00	10,82	11,00	11,00	11,00	10,96	10,81	10,97	10,97	11,00	11,00	11,00	10,36	10,97	11,00	11,00	10,97	10,97	11,00	10,97	10,96	4,65	10,95	4,72	11,00	11,00	11,00	11,00	10,97	11,00	10,97	10,97	10,97	10,97	11,00	11,00	11,00
DENV-3	11,00	10,98	11,00	10,99	11,00	11,00	10,99	11,00	11,00	11,00	11,00	11,00	11,00	11,00	11,00	11,00	10,99	11,00	11,00	11,00	10,99	11,00	11,00	11,00	11,00	11,00	11,00	11,00	11,00	11,00	11,00	11,00	11,00	10,99	11,00	11,00	10,20	11,00	10,85	11,00	11,00	11,00	11,00	11,00	11,00	11,00	11,00	11,00	11,00	11,00
DENV-4	11,00	11,00	11,00	11,00	11,00	11,00	11,00	10,87	11,00	11,00	11,00	10,83	11,00	10,73	11,00	11,00	11,00	11,00	11,00	11,00	11,00	11,00	11,00	10,89	11,00	11,00	11,00	11,00	11,00	11,00	11,00	11,00	10,83	10,78	9,15	11,00	11,00	11,00	11,00	11,00	11,00	11,00	11,00	11,00	11,00	11,00	11,00	11,00	11,00	11,00

aa	351	352	353	354	355	356	357	358	359	360	361	362	363	364	365	366	367	368	369	370	371	372	373	374	375	376	377	378	379	380	381	382	383	384	385	386	387	388	389	390	391	392	393	394	395	396	397	398	399	400
DENV-1	10,96	10,99	11,00	11,00	10,99	10,99	11,00	11,00	11,00	11,00	11,00	11,00	11,00	11,00	8,33	8,71	10,99	10,93	11,00	11,00	6,73	11,00	10,96	11,00	10,97	10,98	11,00	11,00	8,46	11,00	10,99	10,97	10,05	10,99	11,00	10,95	11,00	10,91	10,99	10,93	11,00	11,00	10,82	10,98	11,00	11,00	10,99	11,00	10,98	10,83
DENV-2	11,00	11,00	11,00	11,00	11,00	11,00	11,00	11,00	10,96	11,00	11,00	11,00	10,96	10,97	10,98	11,00	11,00	11,00	10,97	11,00	11,00	10,97	10,98	11,00	9,48	10,98	10,97	10,97	10,42	11,00	11,00	10,95	10,90	11,00	11,00	10,97	10,85	8,69	10,96	10,19	10,96	11,00	10,76	10,96	10,98	11,00	10,89	10,98	11,00	10,41
DENV-3	10,99	11,00	11,00	11,00	11,00	10,98	11,00	11,00	11,00	11,00	11,00	11,00	10,99	11,00	10,81	8,74	10,99	10,95	10,99	10,99	11,00	10,10	10,81	11,00	8,14	11,00	11,00	10,99	10,93	11,00	11,00	11,00	10,97	10,99	10,98	10,99	11,00	11,00	10,97	10,37	11,00	11,00	11,00	11,00	10,96	11,00	11,00	11,00	11,00	10,87
DENV-4	11,00	11,00	11,00	10,78	11,00	11,00	11,00	10,86	10,78	10,78	11,00	10,81	11,00	11,00	10,69	10,86	10,79	11,00	10,62	10,86	10,89	8,21	8,65	11,00	10,88	10,34	11,00	11,00	10,88	11,00	11,00	11,00	10,13	10,90	10,73	11,00	10,09	10,83	11,00	5,94	11,00	11,00	11,00	11,00	11,00	10,56	11,00	11,00	11,00	10,95

aa	401	402	403	404	405	406	407	408	409	410	411	412	413	414	415	416	417	418	419	420	421	422	423	424	425	426	427	428	429	430	431	432	433	434	435	436	437	438	439	440	441	442	443	444	445	446	447	448	449	450
DENV-1	10,96	11,00	10,99	11,00	11,00	10,98	10,97	10,98	10,93	11,00	11,00	10,97	10,96	10,50	10,98	11,00	10,96	10,99	11,00	10,99	10,96	10,96	10,98	10,98	10,98	10,94	10,99	11,00	10,58	10,92	10,94	11,00	10,89	11,00	10,91	8,82	9,96	11,00	10,98	11,00	11,00	11,00	11,00	10,99	11,00	10,99	10,98	10,96	10,97	10,97
DENV-2	9,81	11,00	11,00	11,00	10,95	11,00	11,00	11,00	10,99	11,00	11,00	10,39	10,99	10,98	10,92	10,97	10,98	10,96	11,00	10,97	11,00	11,00	10,99	10,97	10,98	11,00	11,00	11,00	3,24	2,70	11,00	10,96	10,91	11,00	10,39	10,97	7,57	11,00	11,00	10,71	10,98	11,00	11,00	10,97	11,00	11,00	11,00	11,00	10,96	11,00
DENV-3	11,00	11,00	10,98	11,00	10,96	10,98	10,98	10,99	11,00	11,00	11,00	11,00	11,00	11,00	10,99	10,99	10,98	11,00	11,00	10,94	10,98	11,00	10,24	10,98	11,00	11,00	11,00	11,00	11,00	10,08	11,00	11,00	10,61	10,99	10,98	10,92	10,90	11,00	10,98	10,98	10,98	11,00	10,99	10,86	11,00	11,00	11,00	11,00	11,00	11,00
DENV-4	10,88	11,00	11,00	11,00	10,64	10,86	11,00	11,00	10,92	11,00	11,00	10,95	10,56	11,00	11,00	11,00	11,00	10,83	11,00	10,72	10,88	10,87	9,95	11,00	11,00	11,00	10,18	11,00	10,83	11,00	11,00	11,00	10,88	11,00	11,00	10,84	10,94	11,00	11,00	10,74	11,00	11,00	10,81	11,00	11,00	10,95	11,00	11,00	11,00	11,00

aa	451	452	453	454	455	456	457	458	459	460	461	462	463	464	465	466	467	468	469	470	471	472	473	474	475	476	477	478	479	480	481	482	483	484	485	486	487	488	489	490	491	492	493	494	495	496	497	498	499	500
DENV-1	10,94	10,97	11,00	10,95	11,00	11,00	11,00	11,00	10,98	10,99	11,00	11,00	11,00	11,00	11,00	11,00	11,00	10,98	11,00	11,00	11,00	11,00	10,95	11,00	11,00	11,00	11,00	11,00	11,00	11,00	11,00	11,00	11,00	11,00	11,00	11,00	11,00	11,00	11,00	11,00	11,00	10,96	11,00	11,00	11,00	11,00	11,00	10,98	10,95	11,00
DENV-2	10,97	11,00	11,00	11,00	11,00	11,00	11,00	11,00	11,00	10,98	11,00	9,84	11,00	11,00	11,00	11,00	10,96	11,00	10,99	10,97	11,00	11,00	10,97	10,99	11,00	11,00	11,00	11,00	11,00	11,00	11,00	11,00	10,99	11,00	11,00	11,00	11,00	11,00	11,00	11,00	11,00	11,00	11,00	11,00	11,00	11,00	11,00	11,00	11,00	11,00
DENV-3	11,00	11,00	11,00	11,00	11,00	11,00	10,98	11,00	10,99	11,00	11,00	10,98	11,00	11,00	11,00	11,00	10,98	11,00	10,98	10,99	11,00	11,00	11,00	11,00	11,00	11,00	10,99	11,00	11,00	11,00	10,98	11,00	10,96	11,00	11,00	11,00	11,00	11,00	10,99	10,99	11,00	11,00	10,98	11,00	10,98	11,00	11,00	11,00	11,00	11,00
DENV-4	11,00	11,00	11,00	10,78	11,00	10,78	10,75	11,00	11,00	11,00	11,00	11,00	11,00	11,00	11,00	11,00	11,00	11,00	11,00	11,00	11,00	11,00	11,00	10,92	11,00	11,00	11,00	11,00	11,00	11,00	11,00	11,00	11,00	11,00	11,00	11,00	11,00	11,00	11,00	11,00	11,00	11,00	11,00	11,00	11,00	11,00	11,00	11,00	6,55	11,00

aa	501	502	503	504	505	506	507	508	509	510	511	512	513	514	515	516	517	518	519	520	521	522	523	524	525	526	527	528	529	530	531	532	533	534	535	536	537	538	539	540	541	542	543	544	545	546	547	548	549	550
DENV-1	10,94	11,00	11,00	10,63	11,00	11,00	11,00	11,00	11,00	10,99	10,98	11,00	10,97	10,96	11,00	11,00	11,00	11,00	11,00	11,00	10,98	11,00	10,98	10,29	11,00	10,19	11,00	11,00	10,93	10,96	11,00	11,00	10,99	11,00	11,00	10,99	11,00	11,00	11,00	11,00	11,00	10,83	11,00	10,97	10,96	11,00	10,96	10,93	11,00	11,00
DENV-2	0,48	11,00	11,00	11,00	11,00	11,00	11,00	11,00	11,00	10,99	11,00	11,00	10,93	7,71	10,94	11,00	11,00	11,00	11,00	10,94	9,44	10,98	3,90	10,62	10,99	10,82	10,85	10,97	10,25	10,97	10,91	11,00	10,97	11,00	10,98	11,00	11,00	10,94	10,93	11,00	10,97	10,99	11,00	10,66	10,97	10,97	11,00	10,95	10,95	10,97
DENV-3	10,96	11,00	11,00	11,00	11,00	11,00	11,00	11,00	11,00	11,00	11,00	11,00	10,96	10,99	11,00	11,00	11,00	11,00	11,00	11,00	10,98	11,00	11,00	10,95	10,95	11,00	11,00	10,99	10,98	10,99	10,98	10,98	11,00	11,00	11,00	11,00	11,00	11,00	11,00	11,00	10,98	10,96	11,00	11,00	11,00	11,00	11,00	10,97	10,98	10,99
DENV-4	11,00	10,86	11,00	10,85	11,00	11,00	11,00	11,00	11,00	11,00	11,00	11,00	11,00	11,00	11,00	11,00	11,00	10,95	11,00	11,00	8,31	10,85	10,88	10,83	10,47	10,84	10,84	10,84	11,00	6,96	10,97	11,00	11,00	11,00	11,00	11,00	11,00	11,00	11,00	11,00	10,95	11,00	10,87	11,00	10,94	11,00	11,00	0,00	10,87	11,00

aa	551	552	553	554	555	556	557	558	559	560	561	562	563	564	565	566	567	568	569	570	571	572	573	574	575	576	577	578	579	580	581	582	583	584	585	586	587	588	589	590	591	592	593	594	595	596	597	598	599	600
DENV-1	10,96	8,67	10,99	10,97	10,36	10,91	11,00	10,94	10,62	10,84	11,00	11,00	10,10	11,00	11,00	0,001	7,73	10,99	11,00	10,94	11,00	11,00	11,00	11,00	10,95	10,97	11,00	11,00	11,00	11,00	10,99	10,95	11,00	10,75	10,85	8,30	11,00	10,96	11,00	11,00	10,99	10,95	11,00	11,00	10,97	11,00	10,96	11,00	10,99	10,98
DENV-2	10,88	10,80	10,68	10,87	10,76	10,77	10,98	1,96	10,96	10,79	10,94	10,78	10,76	10,97	10,93	10,86	10,95	10,87	11,00	9,34	10,96	10,97	10,97	10,97	10,96	10,99	10,96	11,00	10,93	10,98	11,00	10,97	10,97	10,96	10,25	10,84	11,00	10,92	10,98	10,98	11,00	10,97	11,00	10,93	11,00	9,28	10,95	10,98	10,97	10,97
DENV-3	10,99	11,00	11,00	10,93	10,95	10,97	10,98	10,83	10,98	10,97	11,00	10,94	10,07	11,00	11,00	10,75	11,00	10,99	11,00	11,00	11,00	11,00	10,98	11,00	11,00	11,00	11,00	11,00	11,00	11,00	11,00	11,00	11,00	11,00	10,99	8,23	11,00	10,99	11,00	11,00	11,00	11,00	11,00	11,00	11,00	11,00	10,99	11,00	11,00	11,00
DENV-4	10,87	10,84	10,79	10,72	10,84	10,84	11,00	10,88	10,88	10,85	10,80	10,79	6,16	10,84	11,00	10,67	11,00	10,75	11,00	11,00	11,00	11,00	11,00	10,89	10,64	11,00	11,00	10,89	11,00	11,00	11,00	11,00	10,84	10,81	10,84	8,17	11,00	11,00	10,75	11,00	11,00	11,00	11,00	11,00	10,95	10,95	11,00	11,00	11,00	11,00

aa	601	602	603	604	605	606	607	608	609	610	611	612		613	614	615	616	617	618	619	620	621	622	623	624	625	626	627	628	629	630	631	632	633	634	635	636		637	638	639	640	641	642	643	644	645	646	647	648
DENV-1	11,00	11,00	11,00	11,00	11,00	11,00	11,00	10,96	11,00	10,98	11,00	11,00		10,97	10,98	10,94	10,93	4,36	11,00	11,00	10,97	11,00	10,94	11,00	10,99	10,95	10,98	10,24	10,96	11,00	0,00	10,96	9,81	10,99	10,99	10,92	8,16	8,47	10,81	11,00		11,00	5,11	10,89	8,25	10,85	10,54	11,00	10,99	9,86
DENV-2	11,00	10,80	11,00	11,00	0,00	11,00	11,00	11,00	11,00	10,85	11,00	11,00		11,00	11,00	10,95	8,64	10,98	10,97	10,94	11,00	11,00	11,00	11,00	11,00	10,97	11,00	10,82	7,43	11,00	10,88	5,14	10,91	10,83	6,72	10,93	10,95		1,73	7,45	11,00	10,97	7,40	10,67	10,83	4,79	9,08	11,00	11,00	3,55
DENV-3	11,00	11,00	11,00	10,98	10,99	11,00	11,00	10,99	11,00	11,00	11,00	10,99	0,00	10,99	11,00	11,00	10,99	10,78	11,00	10,99	10,26	10,98	11,00	11,00	11,00	11,00	11,00	10,95	11,00	11,00	10,68	10,89	10,31	11,00	11,00	11,00	10,91	10,85	10,88	10,91	8,34	10,96	11,00	10,87	10,92	10,84	10,99	11,00	11,00	11,00
DENV-4	11,00	11,00	11,00	11,00	11,00	11,00	11,00	10,58	10,92	11,00	11,00	11,00		11,00	11,00	11,00	11,00	11,00	11,00	11,00	11,00	11,00	11,00	11,00	11,00	11,00	11,00	11,00	10,73	10,90	11,00	8,63	10,95	10,86	11,00	7,88	10,90	10,83	10,51	10,88	10,90	10,57	10,70	10,90	11,00	10,76	10,39	11,00	11,00	7,09

aa	649	650	651	652	653	654	655	656	657	658	659	660	661	662	663	664	665	666	667	668	669	670	671	672	673	674	675	676	677	678	679	680	681	682	683	684	685	686	687	688	689	690	691	692	693	694	695	696	697	698
DENV-1	10,98	7,87	11,00	5,06	10,91	11,00	11,00	10,88	10,97	10,99	10,96	11,00	11,00	11,00	11,00	11,00	11,00	10,94	10,95	10,98	11,00	8,37	10,97	10,96	11,00	11,00	11,00	10,74	10,98	11,00	9,76	10,98	11,00	11,00	10,98	11,00	11,00	11,00	10,96	11,00	11,00	11,00	10,83	10,94	11,00	10,98	11,00	11,00	11,00	10,99
DENV-2	10,71	10,85	11,00	10,96	10,99	10,99	10,96	10,75	11,00	11,00	10,87	10,99	11,00	11,00	11,00	11,00	10,93	10,95	10,97	11,00	11,00	5,09	11,00	10,94	11,00	11,00	10,97	3,61	10,97	11,00	10,90	10,97	11,00	11,00	10,97	11,00	10,97	11,00	8,70	11,00	11,00	10,96	10,97	10,98	11,00	11,00	10,98	10,98	11,00	8,95
DENV-3	10,81	10,96	11,00	11,00	10,98	10,99	10,98	10,78	11,00	11,00	11,00	11,00	10,99	11,00	10,98	11,00	11,00	11,00	10,99	11,00	11,00	10,99	11,00	11,00	11,00	11,00	10,97	10,90	11,00	11,00	10,94	11,00	11,00	11,00	11,00	10,97	11,00	11,00	10,98	11,00	11,00	11,00	10,99	11,00	11,00	11,00	11,00	11,00	11,00	10,99
DENV-4	10,90	10,53	11,00	10,78	11,00	11,00	11,00	10,48	10,89	10,78	10,54	10,95	10,78	10,81	10,75	11,00	11,00	11,00	11,00	11,00	11,00	10,78	11,00	11,00	11,00	11,00	3,96	10,58	10,88	11,00	10,81	11,00	11,00	11,00	11,00	11,00	11,00	11,00	10,86	11,00	11,00	10,78	11,00	11,00	11,00	11,00	11,00	11,00	11,00	11,00

aa	699	700	701	702	703	704	705	706	707	708	709	710	711	712	713	714	715	716	717	718	719	720	721	722	723	724	725	726	727	728	729	730	731	732	733	734	735	736	737	738	739	740	741	742	743	744	745	746	747	748
DENV-1	11,00	11,00	10,98	10,99	11,00	10,98	10,96	10,95	10,98	10,96	10,93	11,00	11,00	11,00	10,97	10,98	11,00	11,00	10,94	11,00	11,00	11,00	10,97	11,00	10,96	10,98	11,00	11,00	11,00	11,00	11,00	10,95	10,99	11,00	10,98	11,00	10,94	11,00	11,00	11,00	11,00	11,00	11,00	10,97	11,00	11,00	11,00	11,00	10,91	11,00
DENV-2	10,97	11,00	10,82	11,00	11,00	11,00	10,97	11,00	11,00	11,00	11,00	11,00	11,00	11,00	11,00	11,00	10,98	10,86	7,54	11,00	10,90	11,00	11,00	11,00	10,06	11,00	10,97	10,95	10,95	11,00	11,00	10,85	11,00	10,98	10,96	11,00	10,98	10,95	10,99	11,00	11,00	10,97	11,00	11,00	10,97	10,97	11,00	11,00	10,86	10,95
DENV-3	11,00	10,98	10,98	11,00	11,00	11,00	11,00	11,00	11,00	11,00	11,00	11,00	11,00	11,00	11,00	11,00	11,00	11,00	11,00	11,00	11,00	11,00	11,00	11,00	10,97	11,00	11,00	11,00	11,00	11,00	11,00	10,99	11,00	10,99	11,00	11,00	10,99	11,00	11,00	11,00	11,00	11,00	11,00	11,00	11,00	10,94	11,00	11,00	11,00	10,99
DENV-4	11,00	11,00	11,00	10,79	11,00	11,00	11,00	11,00	11,00	11,00	11,00	11,00	11,00	11,00	11,00	11,00	10,94	10,90	10,73	11,00	11,00	11,00	11,00	11,00	10,88	11,00	10,71	11,00	11,00	11,00	11,00	10,86	10,66	11,00	11,00	11,00	11,00	11,00	11,00	11,00	11,00	11,00	11,00	11,00	11,00	11,00	11,00	11,00	10,72	11,00

aa	749	750	751	752	753	754	755	756	757	758	759	760	761	762	763	764	765	766	767	768	769	770	771	772	773	774	775	776	777	778	779	780	781	782	783	784	785	786	787	788	789	790	791	792	793	794	795	796	797	798
DENV-1	10,95	10,98	10,97	11,00	10,98	11,00	10,98	11,00	11,00	11,00	11,00	11,00	11,00	10,92	11,00	11,00	11,00	10,97	11,00	10,98	10,99	11,00	10,98	11,00	11,00	11,00	11,00	10,96	10,98	10,98	10,91	10,92	11,00	11,00	10,75	10,98	10,77	9,24	11,00	8,63	11,00	10,63	10,93	11,00	11,00	11,00	10,98	11,00	11,00	11,00
DENV-2	7,42	10,94	11,00	11,00	11,00	10,96	11,00	10,97	10,98	11,00	11,00	11,00	10,96	11,00	2,24	10,96	10,91	10,97	11,00	10,95	10,95	10,97	10,97	11,00	11,00	11,00	10,97	11,00	11,00	10,87	11,00	11,00	11,00	10,98	10,97	10,97	10,37	11,00	11,00	10,22	11,00	10,84	11,00	11,00	11,00	11,00	10,97	10,96	10,97	11,00
DENV-3	10,04	11,00	11,00	11,00	10,99	11,00	10,99	11,00	11,00	11,00	11,00	11,00	11,00	11,00	10,18	11,00	11,00	10,98	10,99	11,00	11,00	11,00	11,00	11,00	11,00	11,00	11,00	11,00	11,00	11,00	11,00	11,00	11,00	11,00	10,96	11,00	10,96	11,00	11,00	10,60	10,99	10,98	11,00	11,00	10,99	11,00	11,00	11,00	11,00	11,00
DENV-4	10,67	11,00	11,00	11,00	11,00	10,78	10,86	11,00	10,88	11,00	11,00	10,83	10,75	10,75	10,66	11,00	11,00	11,00	11,00	11,00	11,00	11,00	11,00	11,00	11,00	10,89	10,89	11,00	10,78	11,00	11,00	11,00	11,00	11,00	11,00	11,00	10,13	10,83	11,00	10,74	11,00	10,86	11,00	11,00	11,00	11,00	11,00	11,00	11,00	11,00

aa	799	800	801	802	803	804	805	806	807	808	809	810	811	812	813	814	815	816	817	818	819	820	821	822	823	824	825	826	827	828	829	830	831	832	833	834	835	836	837	838	839	840	841	842	843	844	845	846	847	848
DENV-1	11,00	11,00	10,99	10,99	11,00	11,00	11,00	11,00	11,00	10,99	11,00	11,00	11,00	11,00	11,00	11,00	10,99	11,00	11,00	11,00	10,98	11,00	10,99	11,00	11,00	11,00	10,61	10,74	11,00	10,96	10,12	7,95	10,96	10,67	11,00	1,60	8,78	10,98	11,00	11,00	11,00	11,00	10,98	11,00	11,00	10,99	10,99	10,97	10,97	11,00
DENV-2	11,00	0,41	10,98	10,98	11,00	10,98	10,96	10,90	10,74	10,94	11,00	11,00	3,79	11,00	11,00	10,97	10,90	11,00	11,00	10,98	6,76	10,90	11,00	11,00	11,00	10,97	10,99	10,39	11,00	11,00	10,78	11,00	10,94	10,92	11,00	10,99	10,99	7,52	10,97	10,96	11,00	10,97	11,00	11,00	10,97	11,00	11,00	11,00	11,00	11,00
DENV-3	11,00	10,98	11,00	11,00	11,00	11,00	11,00	11,00	10,97	10,97	11,00	10,99	10,83	11,00	11,00	11,00	11,00	11,00	11,00	11,00	11,00	10,98	11,00	11,00	11,00	10,99	10,94	10,98	11,00	10,99	11,00	10,91	10,99	11,00	11,00	10,97	9,27	10,96	11,00	11,00	11,00	11,00	11,00	11,00	11,00	11,00	11,00	11,00	11,00	11,00
DENV-4	11,00	10,78	11,00	11,00	11,00	11,00	11,00	10,88	11,00	10,89	11,00	11,00	10,83	11,00	11,00	11,00	10,94	11,00	11,00	11,00	10,90	10,95	11,00	11,00	10,88	11,00	3,91	11,00	11,00	11,00	11,00	10,42	10,67	11,00	11,00	10,95	10,95	10,90	11,00	11,00	10,89	11,00	11,00	11,00	11,00	11,00	10,81	11,00	11,00	11,00

aa	849	850	851	852	853	854	855	856	857	858	859	860	861	862	863	864	865	866	867	868	869	870	871	872	873	874	875	876	877	878	879	880	881	882	883	884	885	886	887	888	889	890	891	892	893	894	895	896	897	898	899	900
DENV-1	11,00	10,96	11,00	11,00	11,00	10,97	10,93	11,00	11,00	11,00	11,00	10,98	8,60	11,00	10,97	11,00	10,98	11,00	10,98	10,94	10,98	11,00	11,00	10,96	11,00	10,29	11,00	10,97	10,98	10,79	11,00	9,27	10,99	10,97	11,00	10,80	11,00	10,99	10,97	11,00	11,00	11,00	10,91	10,93	7,91	10,47	10,14	10,98	11,00	10,91	10,96	11,00
DENV-2	11,00	10,94	11,00	11,00	11,00	11,00	10,98	10,98	10,96	10,96	10,92	11,00	10,85	11,00	11,00	10,92	6,53	11,00	10,95	10,90	10,98	10,97	10,99	10,95	11,00	10,73	11,00	8,72	11,00	9,05	11,00	10,63	10,99	10,96	10,97	11,00	11,00	11,00	11,00	11,00	11,00	10,80	9,80	11,00	10,98	10,93	10,62	10,90	11,00	10,57	11,00	11,00
DENV-3	11,00	10,98	11,00	11,00	10,98	10,90	11,00	11,00	10,99	11,00	11,00	10,99	11,00	11,00	11,00	8,47	10,98	11,00	11,00	10,98	10,98	10,99	11,00	11,00	11,00	10,98	11,00	9,85	11,00	11,00	11,00	10,94	11,00	11,00	11,00	11,00	10,98	11,00	11,00	11,00	11,00	10,99	11,00	10,96	11,00	11,00	10,61	11,00	11,00	10,95	10,99	11,00
DENV-4	11,00	11,00	11,00	11,00	11,00	10,88	11,00	11,00	11,00	11,00	11,00	11,00	11,00	11,00	10,78	10,60	10,74	10,86	11,00	10,09	10,89	11,00	11,00	11,00	11,00	11,00	11,00	10,51	10,84	10,67	11,00	9,94	11,00	10,89	10,84	8,18	6,68	10,92	10,68	10,84	11,00	10,83	10,90	7,90	0,86	10,90	10,69	11,00	11,00	11,00	11,00	-

Conservation score scale: 0-3 | 4-6 | 7-9 | 10-11

Figure S2. Overall alignment of the full-length residue-specific NS5 conservation scores for each DENV serotype (DENV1-DENV4). A color-coded scheme from red to blue was used to display the different conservation categories: red - highly variable residues (0-3); pink -variable residues (4-6); light blue - conserved residues (7-9); and, dark blue - highly conserved residues (10-11). The conservation score for each residue is displayed below its corresponding position in white, for all serotypes. Residue numbering is based on DENV2 coordinates.
