# Supplementary material for: Dengue Virus NS5 Target Discovery: A Comprehensive in Silico Exploration of Novel Druggable Sites for Pan-Serotype Antiviral Design
Source: Int J Mol Sci. 2026 Jun 22;27(12):5639. doi: 10.3390/ijms27125639 (PMC13299206; doi:10.3390/ijms27125639)
Supplement: Supplementary file 1 [file ijms-27-05639-s001.zip › Figure_S3.pdf]

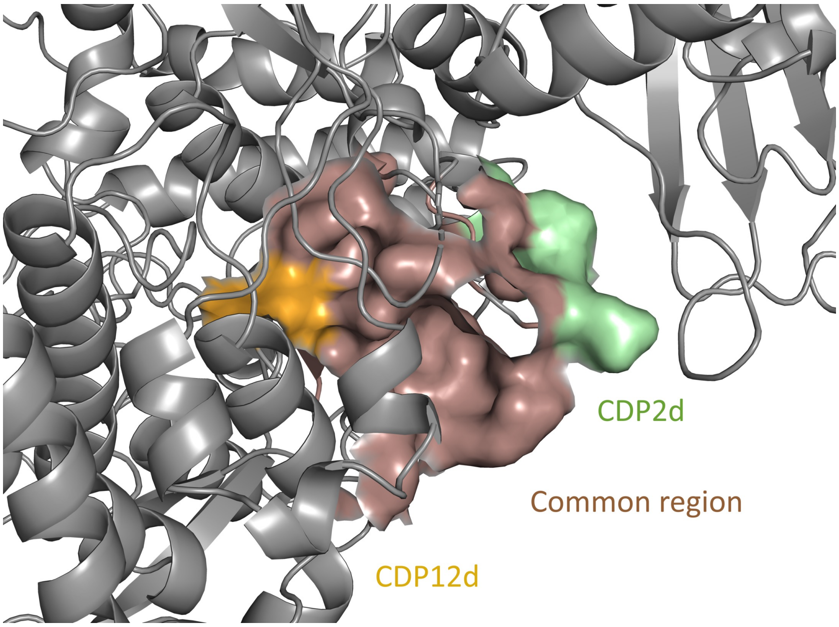

Figure S3. Structural mapping of CDP12d (orange), one of the most promising dimer-associated pockets, and the leading monomer-associated CDP2 (green) onto the DENV NS5 structure (PDB ID: 5ZQK), highlighting their extensive overlapping region (brown).
