# Supplementary material for: Dengue Virus NS5 Target Discovery: A Comprehensive in Silico Exploration of Novel Druggable Sites for Pan-Serotype Antiviral Design
Source: Int J Mol Sci. 2026 Jun 22;27(12):5639. doi: 10.3390/ijms27125639 (PMC13299206; doi:10.3390/ijms27125639)
Supplement: Supplementary file 1 [file ijms-27-05639-s001.zip › Figure_S4.pdf]

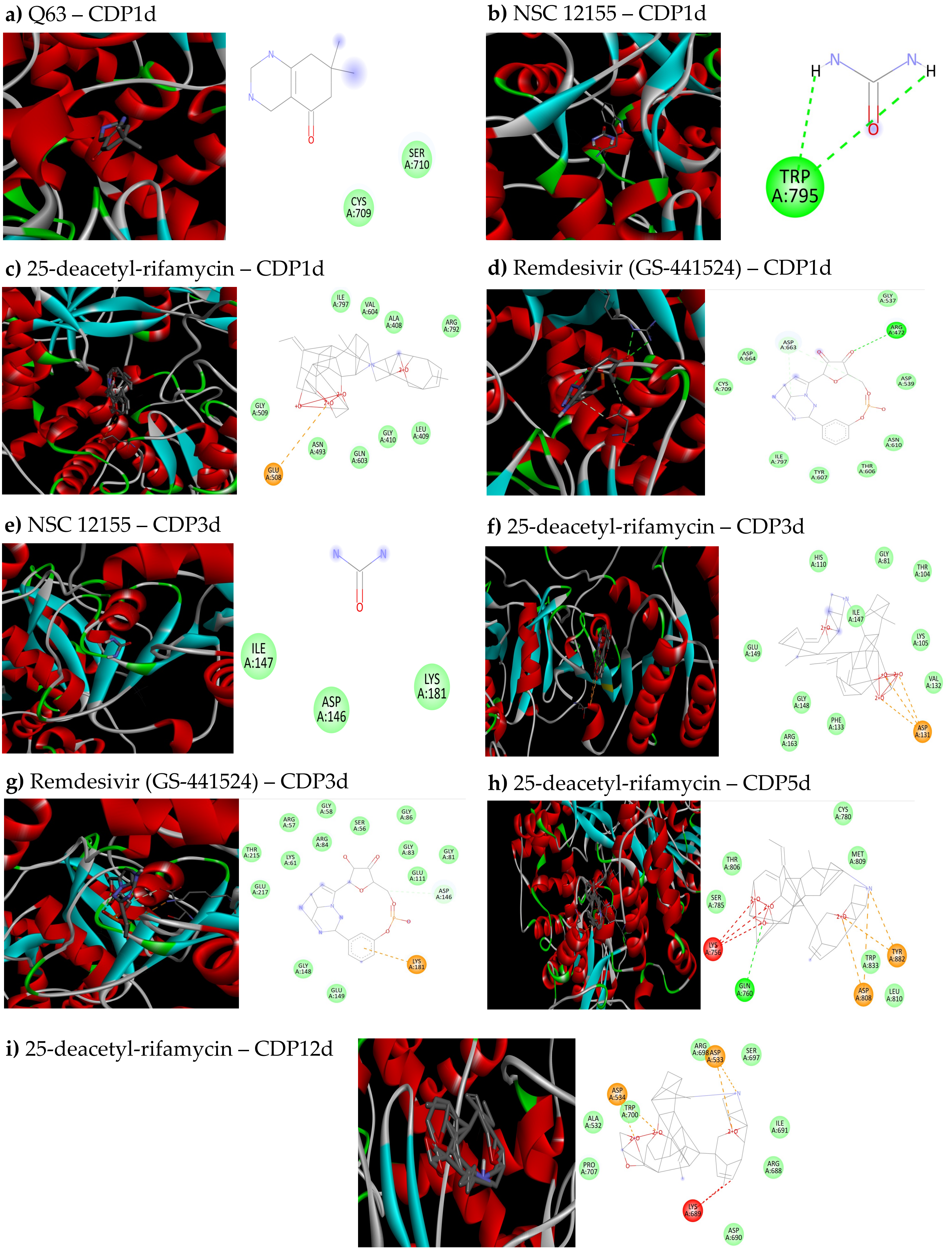

Figure S4. Binding interactions of selected preclinical candidates with the leading CDPds exhibiting affinities below the defined threshold. Key interacting residues are highlighted for each complex. Panels correspond to CDP1d **(a-d)**, CDP3d **(e-g)**, CDP5d **(h)**, and CDP12d **(i)**.
