# Supplementary material for: Dengue Virus NS5 Target Discovery: A Comprehensive in Silico Exploration of Novel Druggable Sites for Pan-Serotype Antiviral Design
Source: Int J Mol Sci. 2026 Jun 22;27(12):5639. doi: 10.3390/ijms27125639 (PMC13299206; doi:10.3390/ijms27125639)
Supplement: Supplementary file 1 [file ijms-27-05639-s001.zip › Table_S1.pdf]

Table S1. Summary of preclinical antiviral candidates with reported activity against DENV NS5.

Compound	Source	Target	Evaluation	Approach used for identification	Interacting residues (DENV2 numbering)	References [a]
Cordycepin	Natural	NS5 MTase + RdRp	*In vitro*	Exploratory phytochemical screening; historically used in Chinese traditional medicine with reported antiviral properties	MTase: G81, G83, T104, K105, H110, E111, F133, I147 (SAM-binding site); RdRp: H711, R729, R737, M761, Y766, T794, W795, S796 (N pocket)	[1–3]
AT-752 (prodrug of AT-9010)	Synthetic	NS5 MTase + RdRp	*In vitro*; *in vivo*; clinic	Drug repurposing; built on prior molecules developed for COVID-19 and HCV	MTase: K14, L17, N18, L20, R22, F25, S150, R212, S214 (GTP-binding site); RdRp: R472, K358, K689	[4–6]
Azidothymidine-based triazoles	Synthetic	NS5 MTase	*In vitro*	Drug repurposing; originally investigated as HIV-1 inhibitors	ND	[1,7]
Compound 10	Synthetic	NS5 MTase	Enzymatic assay	Rationally design to target a cavity in Flavivirus MTase; cavity located next to the SAM-binding site (Benzyl-SAH derivatives)	S56, D79, L80, R84, G86, W87, T104, H110, E111, D131, V132, F133, D146, I147, R163, V164, M167	[1,8]
BG-323	Synthetic	NS5 MTase	Enzymatic + subgenomic replicon assays	High-throughput screening (NS5 RNA capping activity)	ND	[1,9]
NSC 12155	Synthetic	NS5 MTase	*In vitro*	Structure-based virtual screening targeting the SAM-binding site of WNV MTase	G81, T104, K105, H110, E111, K130, D131, V132, F133, D146, I147	[1,10]
Myrtopsis corymbose extracts	Natural	NS5 RdRp	Enzymatic assay	Exploratory phytochemical screening; native plant-derived extracts	ND	[1,11]
RK-0404678	Synthetic	NS5 RdRp	*In vitro*	High-throughput screening (*in vitro* replication assay)	Site 1: S763, R773, N777, C780, D808, M809, W833, Y882, M883; Site 2: V507, E510, G511, S661, C709	[1,12]
Trigocherrins	Natural	NS5 RdRp	*In vitro*	Exploratory phytochemical screening; isolated from native plant-derived extracts (bark and wood of *Trigonostemon cherrieri*)	ND	[1,13]
Trigocherriolides	Natural	NS5 RdRp	*In vitro*	Same as trigocherrins	ND	[1,13]

Chartaceones	Natural	NS5 RdRp	*In vitro*	Exploratory phytochemical screening; isolated from a native plant-derived extract (bark of *Cryptocarya chartacea Kosterm*)	ND	[1,14]
Avicularin	Natural	NS5 RdRp	*In vitro*	Exploratory phytochemical screening; isolated from medicinal plant-derived extracts with reported antiviral activity (flavonoid)	ND	[1,15,16]
Quercitrin	Natural	NS5 RdRp	*In vitro*	Exploratory phytochemical screening; isolated from a medicinal plant-derived extract (leaf extract of *Carpolepis laurifolia*)	ND	[1,16]
Betulinic acid	Natural	NS5 RdRp	*In vitro*	Same as quercitrin	ND	[1,16]
Spiraeoside	Natural	NS5 RdRp	*In vitro*	Exploratory screening using commercial flavonoids (reported antiviral activity)	ND	[1,16]
Rutin	Natural	NS5 RdRp	*In vitro*	Same as spiraeoside	ND	[1,16]
Pyridobenzothiazolones (PBTZ)	Synthetic	NS5 RdRp	*In vitro*	Structure-based virtual screening using a library of published and unpublished compounds designed to target specifically HCV NS5B polymerase	K326, L327, L328, K330, W859, I863, A866 (cavity B) (interacting residues for compound 5)	[1,17,18]
(E)-tridec-2-en-4-ynedioic acid (anacolosine)	Natural	NS5 RdRp	*In vitro*	Exploratory phytochemical screening; isolated from a plant-derived extract (leaves of *Anacolosa pervilleana*)	ND	[1,19]
Octadeca-9,11,13-triynoic acid	Natural	NS5 RdRp	*In vitro*	Same as anacolosine	ND	[1,19]
Octadic-13-en-9,11- diynoic acid	Natural	NS5 RdRp	*In vitro*	Same as anacolosine	ND	[1,19]
Octadic-13-en-11-ynoic acid	Natural	NS5 RdRp	*In vitro*	Same as anacolosine	ND	[1,19]
Flacourtosides	Natural	NS5 RdRp	*In vitro*	Exploratory phytochemical screening; isolated from a plant-derived extract (stem bark of *Flacourtia ramontchi*)	ND	[20,21]

7-deaza-20 -C-methyladenosine (7-DMA)	Synthetic	NS5 RdRp	*In vitro*	Drug repurposing; originally investigated as a potent HCV inhibitor	ND	[1,22]
INX-08189	Synthetic	NS5 RdRp	*In vitro*	Same as 7-DMA	ND	[1,23]
Galidesivir (BCX4430)	Synthetic	NS5 RdRp	*In vitro; in vivo*	Drug repurposing; originally investigated for HCV and Filoviruses (EBOV and Marburg)	Q63, V66, E67, R68, N69, V71, I72, P73, Y90, G93, L94, K95, K96, T291, H293, D295, E297, N298, P299, K301, E310, P348, F349, G350, Q351, Q352, R353, V354, K356, E357, R582, P583, T584, P585, T586, V589	[1,24,25]
Balapiravir (prodrug of R1479)	Synthetic	NS5 RdRp	*In vitro; in vivo*; clinic	Drug repurposing; originally developed for the treatment of HCV infection	ND	[1,26]
NITD008	Synthetic	NS5 RdRp	*In vitro; in vivo*	High-throughput screening focused on adenosine analogues (*in vitro* replication assay)	ND	[1,26,27]
2′-C-methylcytidine (2CMC)	Synthetic	NS5 RdRp	*In vitro; in vivo*	Drug repurposing; originally investigated as a target of HCV NS5B polymerase	ND	[1,28]
Compound 2	Synthetic	NS5 RdRp	*In vitro*	High-throughput screening (Novartis' compound library)	ND	[29]
Compounds 27 and 29 (analogues)	Synthetic	NS5 RdRp	*In vitro*	X-ray-based fragment screening (Novartis' fragment collection) followed by structure-based design targeting DENV NS5 polymerase	R729, M761, M765, T794, W795 (C27), S796, H800, Q802, W803 (N pocket)	[30,31]
16i	Synthetic	NS5 RdRp + host c-Src kinase	*In vitro*	Structure-based virtual screening targeting DENV NS5 cavity B using a library of Src active scaffolds. (dual NS5/Src inhibitors)	L327, K330, D333, W859, N862, I863, A866	[32]
Q63	Synthetic	NS5 RdRp	*In vitro*	Surface plasmon resonance imaging (SPRi) screening for DENV NS5 inhibitors	L512, H711, R729, R737, M761, T794, S796, H798, A799	[33]
Tadalafil	Synthetic	NS5 RdRp	*In vitro; in vivo*	Drug repurposing; originally developed as a phosphodiesterase type 5 (PDE5) inhibitor	T51, H52, Y119, D257, L258, G259, A260, G261, T262, H264, A267, E268, K301, K356, E357, K358, D360, T361, R362, T363, P364, A536, G537, T540, R541,	[25]

					N682, V687, R688, K689, D690, I691, P692, Q693	
Remdesivir	Synthetic	NS5 RdRp	*In vitro*	Drug repurposing; originally developed as a potential treatment for EBOV; clinically approved for COVID-19	G55, S56, V57, R64, L65, E66, P67, P197, G198, K201, T202, K203, E232, E235, A236, Y356, Y396, T399, K400, L401, N418, F419, R420, A421, G422, R423, R462, I463, G464, R465, N466, P467, A468, Q469, E470, D472	[25,34]
Sofosbuvir	Synthetic	NS5 RdRp	*In vitro*	Drug repurposing; clinically approved HCV infections	D533, S600, D663, D664	[35]
I-OMe tyrphostin AG538 (I-OMe-AG238)	Synthetic	NS5 RdRp	*In vitro*	High-throughput screening targeting the NS5-NS3 interaction interface	ND	[36]
Suramin hexasodium (SHS)	Synthetic	NS5 RdRp	*In vitro*	Same as I-OMe-AG238	ND	[36]
Benzothiophene derivatives	Synthetic	NS5 RdRp	*In vitro*	Structure-based virtual screening targeting the conserved Triple-D motif identified at the active site of a Consensus RdRp Flavivirus structure (POLcon)	D534, D663, D664	[37]
Doxorubicin and Rifamycin	Synthetic	NS5 RdRp	*In vitro*	Drug repurposing through virtual screening of FDA-approved drugs (blind docking)	K756, S763, D808, M809, Y832, W833	[38]
5e	Synthetic	NS5 RdRp	*In vitro*	Designing a new series of acyclic purine nucleosides (scaffold with reported antiviral activity)	S135, N136, R150, Q452, E460, S467, Y468, T500	[39]
UPGNUC255 and UPGNUC558	Synthetic	NS5 RdRp	*In vitro*	High-throughput screening focused on nucleoside analogues (*in vitro* replication assay)	ND	[40]

MTase: Methyltransferase; RdRp: RNA-dependent RNA polymerase; COVID-19: Coronavirus disease 2019; HCV: Hepatitis C virus; HIV-1: Human immunodeficiency virus type 1; WNV: West Nile virus; EBOV: Ebola virus; FDA: Food and Drug Administration; ND: Not determined

[a] Table-specific References

1. Lee, M.F.; Wu, Y.S.; Poh, C.L. Molecular Mechanisms of Antiviral Agents against Dengue Virus. *Viruses* **2023**, *15*, 705, doi:10.3390/v15030705.

2. Panya, A.; Songprakhon, P.; Panwong, S.; Jantakee, K.; Kaewkod, T.; Tragoolpua, Y.; Sawasdee, N.; Lee, V.S.; Nimmanpipug, P.; Yenchitsomanus, P. Cordycepin Inhibits Virus Replication in Dengue Virus-Infected Vero Cells. *Molecules* **2021**, *26*, 3118, doi:10.3390/molecules26113118.

3. Songprakhon, P.; Panya, A.; Choomee, K.; Limjindaporn, T.; Noisakran, S.; Tarasuk, M.; Yenchitsomanus, P. Cordycepin Exhibits Both Antiviral and Anti-Inflammatory Effects against Dengue Virus Infection. *iScience* **2024**, *27*, 110711, doi:10.1016/j.isci.2024.110711.

4. Good, S.S.; Shannon, A.; Lin, K.; Moussa, A.; Julander, J.G.; La Colla, P.; Collu, G.; Canard, B.; Sommadossi, J.-P. Evaluation of AT-752, a Double Prodrug of a Guanosine Nucleotide Analog with *In Vitro* and *In Vivo* Activity against Dengue and Other Flaviviruses. *Antimicrob. Agents Chemother.* **2021**, *65*, doi:10.1128/AAC.00988-21.

5. Feracci, M.; Eydoux, C.; Fattorini, V.; Lo Bello, L.; Gauffre, P.; Selisko, B.; Sutto-Ortiz, P.; Shannon, A.; Xia, H.; Shi, P.-Y.; et al. AT-752 Targets Multiple Sites and Activities on the Dengue Virus Replication Enzyme NS5. *Antiviral Res.* **2023**, *212*, 105574, doi:10.1016/j.antiviral.2023.105574.

6. Palanichamy Kala, M.; St. John, A.L.; Rathore, A.P.S. Dengue: Update on Clinically Relevant Therapeutic Strategies and Vaccines. *Curr. Treat. Options Infect. Dis.* **2023**, *15*, 27–52, doi:10.1007/s40506-023-00263-w.

7. Vernekar, S.K. V.; Qiu, L.; Zhang, J.; Kankanala, J.; Li, H.; Geraghty, R.J.; Wang, Z. 5′-Silylated 3′-1,2,3-Triazolyl Thymidine Analogues as Inhibitors of West Nile Virus and Dengue Virus. *J. Med. Chem.* **2015**, *58*, 4016–4028, doi:10.1021/acs.jmedchem.5b00327.

8. Lim, S.P.; Sonntag, L.S.; Noble, C.; Nilar, S.H.; Ng, R.H.; Zou, G.; Monaghan, P.; Chung, K.Y.; Dong, H.; Liu, B.; et al. Small Molecule Inhibitors That Selectively Block Dengue Virus Methyltransferase. *Journal of Biological Chemistry* **2011**, *286*, 6233–6240, doi:10.1074/jbc.M110.179184.

9. Stahla-Beek, H.J.; April, D.G.; Saeedi, B.J.; Hannah, A.M.; Keenan, S.M.; Geiss, B.J. Identification of a Novel Antiviral Inhibitor of the Flavivirus Guanylyltransferase Enzyme. *J. Virol.* **2012**, *86*, 8730–8739, doi:10.1128/JVI.00384-12.

10. Brecher, M.; Chen, H.; Li, Z.; Banavali, N.K.; Jones, S.A.; Zhang, J.; Kramer, L.D.; Li, H. Identification and Characterization of Novel Broad-Spectrum Inhibitors of the Flavivirus Methyltransferase. *ACS Infect. Dis.* **2015**, *1*, 340–349, doi:10.1021/acsinfecdis.5b00070.

11. Coulerie, P.; Maciuk, A.; Lebouvier, N.; Hnawia, E.; Guillemot, J.; Canard, B.; Figadère, B.; Nour, M. Phytochemical Study of Myrtopsis Corymbosa, Perspectives for Anti Dengue Natural Compound Research. *Records of Natural Products* **2013**, *7*, 250–253.

12. Shimizu, H.; Saito, A.; Mikuni, J.; Nakayama, E.E.; Koyama, H.; Honma, T.; Shirouzu, M.; Sekine, S.; Shioda, T. Discovery of a Small Molecule Inhibitor Targeting Dengue Virus NS5 RNA-Dependent RNA Polymerase. *PLoS Negl. Trop. Dis.* **2019**, *13*, e0007894, doi:10.1371/journal.pntd.0007894.

13. Allard, P.-M.; Leyssen, P.; Martin, M.-T.; Bourjot, M.; Dumontet, V.; Eydoux, C.; Guillemot, J.-C.; Canard, B.; Poullain, C.; Guéritte, F.; et al. Antiviral Chlorinated Daphnane Diterpenoid Orthoesters from the Bark and Wood of Trigonostemon Cherrieri. *Phytochemistry* **2012**, *84*, 160–168, doi:10.1016/j.phytochem.2012.07.023.

14. Allard, P.-M.; Dau, E.T.H.; Eydoux, C.; Guillemot, J.-C.; Dumontet, V.; Poullain, C.; Canard, B.; Guéritte, F.; Litaudon, M. Alkylated Flavanones from the Bark of Cryptocarya Chartacea As Dengue Virus NS5 Polymerase Inhibitors. *J. Nat. Prod.* **2011**, *74*, 2446–2453, doi:10.1021/np200715v.

15. Coulerie, P.; Eydoux, C.; Hnawia, E.; Stuhl, L.; Maciuk, A.; Lebouvier, N.; Canard, B.; Figadère, B.; Guillemot, J.-C.; Nour, M. Biflavonoids of Dacrydium Balansae with Potent Inhibitory Activity on Dengue 2 NS5 Polymerase. *Planta Med.* **2012**, *78*, 672–677, doi:10.1055/s-0031-1298355.

16. Coulerie, P.; Maciuk, A.; Eydoux, C.; Hnawia, E.; Lebouvier, N.; Figadère, B.; Guillemot, J.; Nour, M. New Inhibitors of the DENV NS5 RdRp from Carpolepis Laurifolia as Potential Antiviral Drugs for Dengue Treatment. *Records of Natural Products* **2014**, *8*, 286–289.

17. Tarantino, D.; Cannalire, R.; Mastrangelo, E.; Croci, R.; Querat, G.; Barreca, M.L.; Bolognesi, M.; Manfroni, G.; Cecchetti, V.; Milani, M. Targeting Flavivirus RNA Dependent RNA Polymerase through a Pyridobenzothiazole Inhibitor. *Antiviral Res.* **2016**, *134*, 226–235, doi:10.1016/j.antiviral.2016.09.007.

18. Cannalire, R.; Ki Chan, K.W.; Burali, M.S.; Gwee, C.P.; Wang, S.; Astolfi, A.; Massari, S.; Sabatini, S.; Tabarrini, O.; Mastrangelo, E.; et al. Pyridobenzothiazolones Exert Potent Anti-Dengue Activity by Hampering Multiple Functions of NS5 Polymerase. *ACS Med. Chem. Lett.* **2020**, *11*, 773–782, doi:10.1021/acsmedchemlett.9b00619.

19. Bourjot, M.; Leyssen, P.; Eydoux, C.; Guillemot, J.-C.; Canard, B.; Rasoanaivo, P.; Guéritte, F.; Litaudon, M. Chemical Constituents of Anacolosa Pervilleana and Their Antiviral Activities. *Fitoterapia* **2012**, *83*, 1076–1080, doi:10.1016/j.fitote.2012.05.004.

20. Bourjot, M.; Leyssen, P.; Eydoux, C.; Guillemot, J.-C.; Canard, B.; Rasoanaivo, P.; Guéritte, F.; Litaudon, M. Flacourtosides A–F, Phenolic Glycosides Isolated from *Flacourtia Ramontchi*. *J. Nat. Prod.* **2012**, *75*, 752–758, doi:10.1021/np300059n.

21. Rakotoarimanana, A.K.; Carriere Richez, P.; Vestalys Ramanandraibe, V.; Bialecki, A.; El Kalamouni, C. Antiviral Strategies Against Dengue Virus: Recent Insights into Compounds Targeting Viral and Host Factors. In *Dengue Virus Evolution: From Emergence to a Global Health Crisis. Current Topics in Microbiology and Immunology*; Duarte dos Santos, C.N., Giovanetti, M., Eds.; Springer: Cham, Switzerland, 2025; Vol. 447, pp. 287–335.

22. Olsen, D.B.; Eldrup, A.B.; Bartholomew, L.; Bhat, B.; Bosserman, M.R.; Ceccacci, A.; Colwell, L.F.; Fay, J.F.; Flores, O.A.; Getty, K.L.; et al. A 7-Deaza-Adenosine Analog Is a Potent and Selective Inhibitor of Hepatitis C Virus Replication with Excellent Pharmacokinetic Properties. *Antimicrob. Agents Chemother.* **2004**, *48*, 3944–3953, doi:10.1128/AAC.48.10.3944-3953.2004.

23. Yeo, K.L.; Chen, Y.-L.; Xu, H.Y.; Dong, H.; Wang, Q.-Y.; Yokokawa, F.; Shi, P.-Y. Synergistic Suppression of Dengue Virus Replication Using a Combination of Nucleoside Analogs and Nucleoside Synthesis Inhibitors. *Antimicrob. Agents Chemother.* **2015**, *59*, 2086–2093, doi:10.1128/AAC.04779-14.

24. Warren, T.K.; Wells, J.; Panchal, R.G.; Stuthman, K.S.; Garza, N.L.; Van Tongeren, S.A.; Dong, L.; Retterer, C.J.; Eaton, B.P.; Pegoraro, G.; et al. Protection against Filovirus Diseases by a Novel Broad-Spectrum Nucleoside Analogue BCX4430. *Nature* **2014**, *508*, 402–405, doi:10.1038/nature13027.

25. Rashid, M.H.O.; Ezzikouri, S.; Soliman, A.M.; Akter, L.; Momohara, K.; Hifumi, T.; Miyoshi, N.; Hishiki, T.; Abdel-Moneim, A.S.; Kohara, M.; et al. Drug Repositioning: Identification of Potent Inhibitors of NS3 Protease and NS5 RdRp for Control of DENV Infection. *Biomedicine & Pharmacotherapy* **2025**, *187*, 118104, doi:10.1016/j.biopha.2025.118104.

26. Chen, Y.-L.; Abdul Ghafar, N.; Karuna, R.; Fu, Y.; Lim, S.P.; Schul, W.; Gu, F.; Herve, M.; Yokohama, F.; Wang, G.; et al. Activation of Peripheral Blood Mononuclear Cells by Dengue Virus Infection Depotentiates Balapiravir. *J. Virol.* **2014**, *88*, 1740–1747, doi:10.1128/JVI.02841-13.

27. Yin, Z.; Chen, Y.-L.; Schul, W.; Wang, Q.-Y.; Gu, F.; Duraiswamy, J.; Kondreddi, R.R.; Niyomrattanakit, P.; Lakshminarayana, S.B.; Goh, A.; et al. An Adenosine Nucleoside Inhibitor of Dengue Virus. *Proceedings of the National Academy of Sciences* **2009**, *106*, 20435–20439, doi:10.1073/pnas.0907010106.

28. Lee, J.-C.; Tseng, C.-K.; Wu, Y.-H.; Kaushik-Basu, N.; Lin, C.-K.; Chen, W.-C.; Wu, H.-N. Characterization of the Activity of 2′-C-Methylcytidine against Dengue Virus Replication. *Antiviral Res.* **2015**, *116*, 1–9, doi:10.1016/j.antiviral.2015.01.002.

29. Yin, Z.; Chen, Y.-L.; Kondreddi, R.R.; Chan, W.L.; Wang, G.; Ng, R.H.; Lim, J.Y.H.; Lee, W.Y.; Jeyaraj, D.A.; Niyomrattanakit, P.; et al. N-Sulfonylanthranilic Acid Derivatives as Allosteric Inhibitors of Dengue Viral RNA-Dependent RNA Polymerase. *J. Med. Chem.* **2009**, *52*, 7934–7937, doi:10.1021/jm901044z.

30. Yokokawa, F.; Nilar, S.; Noble, C.G.; Lim, S.P.; Rao, R.; Tania, S.; Wang, G.; Lee, G.; Hunziker, J.; Karuna, R.; et al. Discovery of Potent Non-Nucleoside Inhibitors of Dengue Viral RNA-Dependent RNA Polymerase from a Fragment Hit Using Structure-Based Drug Design. *J. Med. Chem.* **2016**, *59*, 3935–3952, doi:10.1021/acs.jmedchem.6b00143.

31. Lim, S.P.; Noble, C.G.; Seh, C.C.; Soh, T.S.; El Sahili, A.; Chan, G.K.Y.; Lescar, J.; Arora, R.; Benson, T.; Nilar, S.; et al. Potent Allosteric Dengue Virus NS5 Polymerase Inhibitors: Mechanism of Action and Resistance Profiling. *PLoS Pathog.* **2016**, *12*, e1005737, doi:10.1371/journal.ppat.1005737.

32. Vincetti, P.; Caporuscio, F.; Kaptein, S.; Gioiello, A.; Mancino, V.; Suzuki, Y.; Yamamoto, N.; Crespan, E.; Lossani, A.; Maga, G.; et al. Discovery of Multitarget Antivirals Acting on Both the Dengue Virus NS5-NS3 Interaction and the Host Src/Fyn Kinases. *J. Med. Chem.* **2015**, *58*, 4964–4975, doi:10.1021/acs.jmedchem.5b00108.

33. Yao, X.; Guo, S.; Wu, W.; Wang, J.; Wu, S.; He, S.; Wan, Y.; Nandakumar, K.S.; Chen, X.; Sun, N.; et al. Q63, a Novel DENV2 RdRp Non-Nucleoside Inhibitor, Inhibited DENV2 Replication and Infection. *J. Pharmacol. Sci.* **2018**, *138*, 247–256, doi:10.1016/j.jphs.2018.06.012.

34. Konkolova, E.; Dejmek, M.; Hřebabecký, H.; Šála, M.; Böserle, J.; Nencka, R.; Boura, E. Remdesivir Triphosphate Can Efficiently Inhibit the RNA-Dependent RNA Polymerase from Various Flaviviruses. *Antiviral Res.* **2020**, *182*, 104899, doi:10.1016/j.antiviral.2020.104899.

35. Xu, H.-T.; Colby-Germinario, S.P.; Hassounah, S.A.; Fogarty, C.; Osman, N.; Palanisamy, N.; Han, Y.; Oliveira, M.; Quan, Y.; Wainberg, M.A. Evaluation of Sofosbuvir (β-D-2′-Deoxy-2′-α-Fluoro-2′-β-C-Methyluridine) as an Inhibitor of Dengue Virus Replication #. *Sci. Rep.* **2017**, *7*, 6345, doi:10.1038/s41598-017-06612-2.

36. Yang, S.N.Y.; Maher, B.; Wang, C.; Wagstaff, K.M.; Fraser, J.E.; Jans, D.A. High Throughput Screening Targeting the Dengue NS3-NS5 Interface Identifies Antivirals against Dengue, Zika and West Nile Viruses. *Cells* **2022**, *11*, 730, doi:10.3390/cells11040730.

37. Wang, L.L.; Karim, S.-U.; Hand, A.; Brunkhorst, R.; Petersen, M.; Altman, S.; Liu, Y.; Zhang, L.; Bai, F.; Xiang, S.-H. Identification of Benzothiophene-Derived Inhibitors of Flaviviruses by Targeting RNA-Dependent RNA Polymerase. *Viruses* **2025**, *17*, 145, doi:10.3390/v17020145.

38. Uzma, A.; Adiba; Kausar, A.; Aslam, M.W.; Ali, L.; Ali, M.; Qazi, A.S.; Haq, A.; Amraiz, D.; Sindi, E.R.; et al. In Silico and in Vitro Antiviral Activity of FDA Approved Drugs from ZINC Database against RNA-Dependent RNA Polymerase of Dengue Virus. *Virol. J.* **2026**, *23*, 76, doi:10.1186/s12985-026-03110-8.

39. Mondal, S.K.; Samantaray, S.; Sahoo, A.S.; Mohapatra, A.; Syed, G.H.; Pal, S. Discovery of Acyclic Purine Nucleosides as Promising Antiviral Agents against Dengue Virus. *Bioorg. Chem.* **2026**, *178*, 109961, doi:10.1016/j.bioorg.2026.109961.

40. Bhakt, P.; Pokharel, S.M.; Li, Y.; Srivastava, T.; Miller, J.; Dittmar, M.; Zhu, Y.; Nguyen, D.; Walter, Z.; Ayyanathan, K.; et al. NS5-Targeting Nucleoside Analogs Inhibit Dengue Virus and Other Flaviviruses. *PLoS Pathog.* **2026**, *22*, e1013970, doi:10.1371/journal.ppat.1013970.
