# Supplementary material for: Dengue Virus NS5 Target Discovery: A Comprehensive in Silico Exploration of Novel Druggable Sites for Pan-Serotype Antiviral Design
Source: Int J Mol Sci. 2026 Jun 22;27(12):5639. doi: 10.3390/ijms27125639 (PMC13299206; doi:10.3390/ijms27125639)
Supplement: Supplementary file 1 [file ijms-27-05639-s001.zip › Table_S2.pdf]

Table S2. Conserved clusters identified along the full-length DENV NS5 protein.

Conserved Clusters	Protein Mapping
**G1 - I33** (33-aa length)	MTase domain - GTP-binding subdomain (residues 17-29)
**E35 - V71** (37-aa length)	MTase domain - Core subdomain (residues 55-71)
**P73 - N175** (103-aa length)	MTase domain - Core subdomain (residues 73-175)
**Q177 - G383** (207-aa length)	MTase domain - Core subdomain (residues 177-222); NS3 protease-interacting site (residues 188-203); C-terminal subdomain (residues 223–263)<br><br>Linker region (264-273)<br><br>RdRp domain - Fingers subdomain (residues 274-316); NS3 helicase-interacting site (residues 320-342)
**L385 - D555** (171-aa length)	RdRp domain - Palm subdomain (residues 498-543); Fingers subdomain (residues 417-497 and 544-555), including the conserved sequence motif A (residues 532-539) and motif F (residues 468-474)
**L564 - T612** (49-aa length)	RdRp domain - Fingers subdomain (residues 564–601); Palm subdomain (residues 602-612), including the conserved sequence motif B (residues 598-612)
**H613 - I629** (17-aa length)	RdRp domain - Palm subdomain (residues 613-629)
**E651 - K722** (72-aa length)	RdRp domain - Palm subdomain, including the conserved sequence motif C (residues 660-664) containing the catalytic G662-D663-D664 (GDD) metal-binding motif, the conserved sequence motif D (residues 681– 689) and the conserved sequence motif E (residues 709–722)
**G724 - G900** (177-aa length)	RdRp domain - Thumb subdomain, including the Priming loop (residues 782-808), the NSL (residues 883–900) and the conserved sequence motif E (residues 724-729)

aa: amino acid; MTase: Methyltransferase; RdRp: RNA dependent RNA Polymerase

Clusters are defined as regions containing at least 10 consecutive residues with a conservation score ≥ 7, based on global conservation scores across the four DENV serotypes. Residue coordinates are based on DENV2 numbering.
