# Supplementary material for: Dengue Virus NS5 Target Discovery: A Comprehensive in Silico Exploration of Novel Druggable Sites for Pan-Serotype Antiviral Design
Source: Int J Mol Sci. 2026 Jun 22;27(12):5639. doi: 10.3390/ijms27125639 (PMC13299206; doi:10.3390/ijms27125639)
Supplement: Supplementary file 1 [file ijms-27-05639-s001.zip › Table_S3.pdf]

Table S3. Top-Ranked Hot Spots (T-RHS) in the DENV NS5-RdRp monomer and full-length NS5 protein dimer conformations.

**NS5-RdRp monomer**	E287, K330, G350, K358, T361, R362, K402, W419, A422, C447, V451, W478, G480, G511, Y531, A532, D533, D534, G537, W538, D539, T543, L571, N575, R595, R596, D597, G602, Q603, T606, M661, I663, D674, K689, R691, W697, E698, V709, F711, V729, N733, L737, G739, R740, R742, C756, G758, S760, Y761, R773, L777, N780, W790, V791, H801, W806, M807, T808, T809, E823, V833, Y882
**Full-length NS5 protein dimer**	L17, N18, F25, S31, E35, V36, D37, R38, A41, E49, H53, V55, S56, R57, G58, K61, D79, L80, G81, C82, G83, R84, G85, G86, W87, Y89, Y90, G93, L94, K95, T104, K105, H110, Y119, V124, D131, V132, F133, D146, I147, G148, E149, S150, R160, R163, V164, L210, S211, S214, H216, M245, R248, D255, V256, D257, R263, E268, P299, Y300, K301, A342, T344, F349, Q351, Q352, R353, V354, F355, K356, K358, V359, T361, R362, T363, K402, V451, N453, E485, F486, E508, G511, M530, Y531, A532, D533, D534, T535, A536, G537, W538, D539, T540, R541, T543, R579, V580, R582, P583, R596, Q598, R599, G600, S601, G602, T606, M661, I663, S664, G665, D666, T682, A683, M687, G688, K689, V690, R691, D693, W697, E698, S700, V709, P710, L737, T754, C756, G758, K759, S760, Y761, R773, L777, N780, V791, T793, Q805, W806, M807, T808, T809, E810, E823, V833, Y882, Y885, M886, P887, S888, M889
