# Supplementary material for: Dengue Virus NS5 Target Discovery: A Comprehensive in Silico Exploration of Novel Druggable Sites for Pan-Serotype Antiviral Design
Source: Int J Mol Sci. 2026 Jun 22;27(12):5639. doi: 10.3390/ijms27125639 (PMC13299206; doi:10.3390/ijms27125639)
Supplement: Supplementary file 1 [file ijms-27-05639-s001.zip › Table_S4.pdf]

Table S4. Characterization of the potential Consensus Druggable Pockets (CDPs) identified in the DENV NS5-RdRp monomer and full-length NS5 dimer conformations.

NS5 Structure	(Sub)Domain	CDP	Drug Score	Size	Residues
RdRp monomer	Thumb Subdomain	CDP1	0,73	17 aa	**756**, 759, **760**, 763, **773**, 776, **777**, **780**, 785, 805, **806**, **807**, **808**, **809**, 810, **833**, **882**
	Palm Subdomain	CDP2	0,68	26 aa	**531**, **532**, **533**, **534**, 535, **538**, 614, 617, 621, **663**, 665, 666, 667, **674**, 681, 682, 686, 687, 688, **689**, **691**, 692, 693, **697**, **698**, 700
	Fingers Subdomain	CDP3	0,65	23 aa	280, 283, 284, **287**, 411, 412, 413, 414, 415, 417, 418, **419**, 434, 448, 449, 450, **451**, 453, 477, **478**, 479, **480**, 579
	Fingers/Palm Subdomains	CDP4	0,64	26 aa	398, 399, **402**, 403, 405, 406, 407, 408, 409, 410, 412, **422**, 423, 479, 482, 483, 485, 486, 493, 494, **602**, **603**, 604, **606**, 607, 794
	Fingers Subdomain	CDP5	0,63	17 aa	357, **358**, 359, **361**, **362**, 363, 364, 365, 366, **543**, 544, 545, **597**, 598, 599, 600, 601
	Thumb Subdomain	CDP6	0,62	22 aa	507, 508, 509, 510, **511**, 512, 513, 514, **661**, **709**, **737**, **742**, **758**, **761**, 764, 765, 792, 793, 800, **801**, 802, 803
	Fingers Subdomain	CDP7	0,43	15 aa	271, 272, 273, 277, 301, 302, 303, 304, **362**, **575**, 577, 594, **595**, **596**, **597**
	Fingers Subdomain	SP1	-	10 aa	288, 309, 311, 314, 413, 415, 455, 579, 581, 590
	Fingers Subdomain	SP2	-	11 aa	345, 346, **350**, 351, 353, 354, 357, 454, 455, 473, 474
Full-length protein dimer	RdRp domain	CDP1d	0,84	68 aa	303, 343, 344, **349**, 350, **351**, **352**, **353**, **354**, **355**, **356**, 357, **358**, **359**, **402**, 403, 406, 407, 408, 409, 410, 411, 412, 454, 455, 456, 457, 458, 459, 460, 461, 469, 470, 471, 472, 473, 474, 475, 476, 477, 478, **535**, **536**, **537**, **538**, **539**, **540**, **579**, **580**, 581, **598**, **599**, **600**, **601**, **602**, 603, 604, **606**, 607, 608, 792, **793**, 794, 795, 796, 797, 798, 799
	MTase/RdRp domains	CDP2d	0,81	32 aa	51, 98, 117, **119**, 121, 122, 123, 125, 260, 261, 262, **263**, 264, 265, 266, 267, **268**, 297, 298, **299**, **300**, **301**, **349**, **352**, **355**, **356**, 360, **582**, **583**, 584, 585, 586
	MTase/RdRp domains	CDP3d	0,73	57 aa	**56**, **57**, **58**, **61**, **79**, **80**, **81**, **82**, **83**, **84**, **85**, **86**, **87**, 103, **104**, **105**, **110**, 111, 130, **131**, **132**, **133**, **146**, **147**, **148**, **149**, **150**, **160**, **163**, **164**, 181, **210**, **211**, 212, **214**, 215, 217, 527, 528, 529, **530**, **531**, 668, 670, 671, 672, 675, **691**, 695, 696, **697**, **698**, 699, **700**, 701, 702, 703
	RdRp domain	CDP4d	0,72	20 aa	**402**, 403, 408, 409, 482, **485**, **486**, 489, 490, 493, 494, 496, 497, 498, **508**, 604, 605, **606**, 607, 608
	RdRp domain	CDP5d	0,65	17 aa	**756**, **759**, **760**, 763, **773**, 776, **777**, **780**, 785, **805**, **806**, **807**, **808**, **809**, **810**, **833**, **882**
	MTase/RdRp domains	CDP6d	0,59	23 aa	155, 158, 159, 162, 187, 188, 189, 621, 622, 625, 626, 642, 645, 646, 649, 650, 654, 669, 670, 671, 673, 674, 676
	RdRp domain	CDP7d	0,57	18 aa	269, 302, **361**, **362**, **363**, 364, 365, 366, 371, **540**, **541**, 542, **543**, 544, 545, **596**, 597, **598**

**Full-length protein dimer (cont.)**	MTase/RdRp domains	CDP8d	0,57	62 aa	**49**, 50, **84**, **89**, **90**, **93**, **94**, 113, 114, 115, 116, 118, **119**, 121, **124**, 125, 126, 130, **131**, **132**, **133**, **146**, **147**, **148**, **149**, **150**, 156, **160**, **163**, **164**, 181, 182, 183, 184, **211**, 212, **214**, 215, **256**, **257**, 258, 317, 345, **349**, **353**, **356**, 357, 527, 528, **531**, 668, 670, 671, 672, 675, 695, 696, **697**, **698**, 699, **700**, 701
	MTase/RdRp domains	CDP9d	0,57	36 aa	130, **131**, **132**, **133**, **146**, **147**, **148**, **149**, **150**, 156, **160**, **163**, **164**, 181, 182, 183, 184, **211**, 212, **214**, 215, 527, 528, **531**, 668, 670, 671, 672, 675, 695, 696, **697**, **698**, 699, **700**, 701
	MTase domain	CDP10d	0,55	14 aa	13, 14, **17**, **18**, **25**, **150**, 151, 157, 183, 184, 186, **214**, 215, **216**
	RdRp domain	CDP11d	0,48	15 aa	283, 287, 413, 415, 434, 437, 438, 448, 449, **451**, **453**, 478, **579**, **580**, 581
	RdRp domain	CDP12d	0,42	25 aa	**531**, **532**, **533**, **534**, **535**, **538**, 614, 618, 621, **663**, **664**, **665**, **666**, 667, 674, 681, 686, **687**, **688**, **689**, **691**, **697**, **698**, **700**, **709**
	MTase/RdRp domains	Pocket-13d	0,38	20 aa	45, 48, **49**, 50, 51, 114, 115, 116, 317, 345, 347, **349**, **353**, **354**, 357, **358**, 467, 468, **582**, **583**
	RdRp domain	Pocket-14d	0,38	17 aa	**361**, **362**, **363**, 364, 365, 366, **540**, **541**, 542, **543**, 544, **598**, **682**, **683**, 684, 685, **687**
	RdRp domain	Pocket-15d	0,36	36 aa	**342**, 343, **344**, **354**, **358**, **359**, 461, 462, 469, **535**, **536**, **537**, **538**, **539**, **540**, **599**, **600**, **601**, **602**, 603, **606**, **661**, 662, **663**, **664**, **689**, **709**, **710**, 711, 729, 733, 734, **737**, **758**, **761**, **793**
	MTase domain	Pocket-16d	0,32	31 aa	**56**, **57**, **58**, **61**, **79**, **80**, **81**, **82**, **83**, **84**, **85**, **86**, **87**, 103, **104**, **105**, **110**, 130, **131**, **132**, **133**, **146**, **147**, **148**, **149**, **150**, **160**, **163**, **164**, **211**, **214**
	MTase domain	Pocket-17d	0,30	22 aa	30, 32, 33, 34, **35**, **36**, **37**, **38**, 39, 42, **57**, 60, **61**, 207, 208, 209, **210**, **211**, 212, 213, 238, 239
	MTase/RdRp domains	SP1d	-	10 aa	**132**, **133**, 134, **163**, 166, 171, 672, 673, 675, 676
	RdRp domain	SP2d	-	10 aa	**532**, **533**, **534**, **688**, **689**, **690**, **691**, **697**, **698**, **700**
	MTase domain	SP3d	-	11 aa	20, 24, 27, 28, **31**, 242, 243, **245**, 246, 247, **248**
	MTase domain	Sp4d	-	10 aa	**37**, **38**, 40, **41**, 52, **53**, 54, **255**, **256**, **257**

MTase: Methyltransferase; RdRp - RNA dependent RNA Polymerase

Top-ranked hot spots (T-RHS) are highlighted in bold.
