# Supplementary material for: Dengue Virus NS5 Target Discovery: A Comprehensive in Silico Exploration of Novel Druggable Sites for Pan-Serotype Antiviral Design
Source: Int J Mol Sci. 2026 Jun 22;27(12):5639. doi: 10.3390/ijms27125639 (PMC13299206; doi:10.3390/ijms27125639)
Supplement: Supplementary file 1 [file ijms-27-05639-s001.zip › Table_S5.pdf]

Table S5. Overview of the key features of DENV NS5 crystallographic structures analyzed in the study, as retrieved from the RCSB Protein Data Bank.

Structure ID	DENV Serotype	Complete protein or specific domain	Number of amino acids	Type of crystallography	Resolution	Year
6IZY	DENV2	RdRp domain	686	X-Ray Diffraction	2.11 Å	2018
6J00	DENV3	RdRp domain	647	X-Ray Diffraction	2.14 Å	2018
5CCV	DENV3	Complete protein	905	X-Ray Diffraction	3.60 Å	2015
5ZQK	DENV2	Complete protein	923	X-Ray Diffraction	2.30 Å	2018

DENV: Dengue Virus; RdRp: RNA dependent RNA Polymerase
